# Supplementary material for: Physical health in young males and risk of chronic musculoskeletal, cardiovascular, and respiratory diseases by middle age: A population-based cohort study
Source: PLoS Med. 2025 Jan 21;22(1):e1004517. doi: 10.1371/journal.pmed.1004517 (PMC11793790; doi:10.1371/journal.pmed.1004517)

**Supplementary material**

**Physical health in young males and risk of chronic musculoskeletal, cardiovascular, and pulmonary diseases by middle age: A population-based cohort study**

Aleksandra Turkiewicz^1*^, Karin Magnusson^1^, Simon Timpka^2^, Ali Kiadaliri^3^, Andrea Dell-Isola^1^, Martin Englund^1^

Aleksandra Turkiewicz

Clinical Epidemiology Unit, Lund University

Remissgatan 4, 222 42 Lund, Sweden

e-mail: [*aleksandra.turkiewicz@med.lu.se*](mailto:aleksandra.turkiewicz@med.lu.se)

**Table of contents**

Table A. Correlations (Pearson’s) between the 7 continuous exposures.

Table B. International Classification of Diseases (ICD)-8 diagnostic codes for identification of diseases at conscription examination.

Table C. Unadjusted risk ratios (95% confidence intervals) for associations between exposure and outcomes. Body mass and height were included in one model.

Table D**.** Associations, risk ratios (95% confidence intervals), between exposures and outcomes adjusted for body mass and height.

Table E**.** Associations, risk ratios (95% confidence intervals), between exposures and outcomes from fully adjusted model, when using body mass index as a measure of body size, instead of height and mass.

Table F. Associations, risk ratios (95% confidence intervals), when removing pulse at rest, cardiorespiratory fitness or both these variables from the fully adjusted model.

Table G. Associations (risk ratios with 95% confidence intervals) between exposures and specific diseases within MSK spectrum.

Methods – Multiple imputation.

Table H. Risk ratios (95% confidence intervals) for associations between exposures and the three chronic diseases from multiply imputed data. All exposures were included in one model. Additionally adjusted for parental occupation and education.

Methods – normalizing cardiorespiratory fitness by body mass.

Table I. The crude risk ratios (RR) with 95% confidence intervals (CI) for associations between cardiorespiratory fitness normalized by body mass (W/kg), with and without main effects of the interaction variables.

Table J. The adjusted risk ratios (RR) with 95% confidence intervals (CI) for associations between cardiorespiratory fitness normalized by body mass (W/kg), with and without main effects of the interaction variables.

Code**.** Stata code for the main analysis model.

Fig A. Risk ratio (95% confidence intervals) estimates with different sets of adjustment. Each outcome is presented in a separate panel.

**Table A**. Pearson’s correlations between the 7 continuous exposures.

|  | Muscle strength | Height | Body mass | Blood pressure | Pulse at rest | Hematocrit |
| --- | --- | --- | --- | --- | --- | --- |
| Height | 0.22 |  |  |  |  |  |
| Body mass | 0.49 | 0.46 |  |  |  |  |
| Blood pressure | 0.12 | 0.05 | 0.20 |  |  |  |
| Pulse at rest | -0.04 | -0.04 | -0.02 | 0.35 |  |  |
| Hematocrit | 0.09 | 0.00 | 0.12 | 0.13 | 0.11 |  |
| Cardiorespiratory fitness | 0.34 | 0.24 | 0.39 | 0.00 | -0.28 | 0.01 |

**Table B.** International Classification of Diseases (ICD)-8 diagnostic codes for identification of diseases at conscription examination.

| **Disease** | **ICD-8 codes** |
| --- | --- |
| Respiratory disease | 493, 519, 491 |
| Cardiovascular disease | 410, 411, 412, 413, 414, 427 |
| Osteoarthritis or back pain | 713, 728 |

**Table C.** Unadjusted risk ratios (95% confidence intervals) for associations between exposure and outcomes. Body mass and height were included in one model.

| Exposure | Musculoskeletal | | Cardiovascular | | Respiratory | |
| --- | --- | --- | --- | --- | --- | --- |
| Mass, per 1SD | 1.17 | [1.14,1.20] | 1.25 | [1.22,1.29] | 1.10 | [1.04,1.17] |
| Height, per 1SD | 0.96 | [0.94,0.99] | 0.90 | [0.87,0.93] | 0.87 | [0.83,0.93] |
| BMI, per 1SD | 1.15 | [1.13,1.17] | 1.23 | [1.20,1.27] | 1.10 | [1.04,1.16] |
| Blood pressure, per 1SD | 0.98 | [0.96,1.00] | 1.13 | [1.09,1.16] | 0.99 | [0.93,1.05] |
| Pulse at rest, per 1SD | 0.92 | [0.90,0.94] | 1.02 | [0.99,1.05] | 1.03 | [0.97,1.10] |
| Hematocrit, per 1SD | 1.03 | [1.00,1.05] | 1.08 | [1.05,1.12] | 1.09 | [1.02,1.16] |
| Muscle factor, per 1SD | 1.14 | [1.12,1.17] | 1.11 | [1.07,1.14] | 1.01 | [0.95,1.07] |
| Cardiorespiratory fitness, per 50 Watt | 1.16 | [1.12,1.20] | 0.95 | [0.90,1.01] | 0.78 | [0.71,0.86] |
| Used drugs | 1.08 | [1.01,1.16] | 1.11 | [1.00,1.22] | 1.43 | [1.20,1.70] |
| Did not use drugs | 1.00 |  | 1.00 |  | 1.00 |  |
| Non smoker | 1.00 |  | 1.00 |  | 1.00 |  |
| 1-5 cigarettes | 0.99 | [0.91,1.07] | 1.12 | [0.99,1.25] | 1.22 | [0.97,1.52] |
| 6-10 cigarettes | 1.06 | [1.00,1.13] | 1.35 | [1.23,1.47] | 1.38 | [1.16,1.64] |
| 11+ cigarettes | 1.22 | [1.16,1.29] | 1.66 | [1.53,1.79] | 2.19 | [1.89,2.54] |
| Alcohol: none | 0.92 | [0.83,1.01] | 0.90 | [0.78,1.05] | 1.10 | [0.85,1.43] |
| Alcohol: 1-100 gram per week | 1.00 |  | 1.00 |  | 1.00 |  |
| Alcohol: 101 or more grams per week | 1.16 | [1.11,1.22] | 1.10 | [1.02,1.18] | 1.32 | [1.15,1.51] |
| Health: Very good | 1.00 |  | 1.00 |  | 1.00 |  |
| Health: Quite good | 0.99 | [0.94,1.04] | 1.03 | [0.96,1.11] | 1.01 | [0.88,1.16] |
| Health: Neither good nor bad | 1.02 | [0.95,1.09] | 1.16 | [1.06,1.27] | 1.57 | [1.33,1.84] |
| Headache: yes, often | 1.41 | [1.26,1.58] | 1.49 | [1.28,1.74] | 1.81 | [1.36,2.41] |
| Headache: yes, sometimes | 1.18 | [1.09,1.26] | 1.19 | [1.08,1.32] | 1.34 | [1.10,1.63] |
| Headache: yes, occasionally | 1.09 | [1.02,1.16] | 1.12 | [1.02,1.23] | 1.11 | [0.93,1.33] |
| Headache: no, never | 1.00 |  | 1.00 |  | 1.00 |  |
| Stomach problems: yes, often | 1.18 | [1.05,1.32] | 1.27 | [1.09,1.48] | 1.51 | [1.15,1.99] |
| Stomach problems: yes, sometimes | 1.12 | [1.05,1.20] | 1.12 | [1.01,1.23] | 1.38 | [1.15,1.65] |
| Stomach problems: yes, occasionally | 1.04 | [0.99,1.09] | 0.98 | [0.92,1.06] | 1.01 | [0.88,1.17] |
| Stomach problems: no, never | 1.00 |  | 1.00 |  | 1.00 |  |

SD – standard deviation

**Table D.** Associations, risk ratios (95% confidence intervals), between exposures and outcomes adjusted for body mass and height.

| Exposure | Musculoskeletal | | Cardiovascular | | Respiratory | |
| --- | --- | --- | --- | --- | --- | --- |
| Blood pressure, per 1SD | 0.95 | [0.92,0.97] | 1.08 | [1.04,1.11] | 0.97 | [0.91,1.04] |
| Pulse at rest, per 1SD | 0.92 | [0.90,0.94] | 1.02 | [0.99,1.05] | 1.03 | [0.97,1.09] |
| Hematocrit, per 1SD | 1.01 | [0.98,1.03] | 1.05 | [1.02,1.09] | 1.07 | [1.01,1.14] |
| Muscle factor, per 1SD | 1.08 | [1.06,1.11] | 1.01 | [0.98,1.05] | 0.98 | [0.92,1.05] |
| Cardiorespiratory fitness, per 50 Watt | 1.09 | [1.04,1.13] | 0.84 | [0.79,0.89] | 0.72 | [0.64,0.81] |
| Used drugs | 1.10 | [1.03,1.18] | 1.14 | [1.03,1.26] | 1.43 | [1.20,1.70] |
| Did not use drugs | 1.00 |  | 1.00 |  | 1.00 |  |
| Non smoker | 1.00 |  | 1.00 |  | 1.00 |  |
| 1-5 cigarettes | 1.01 | [0.93,1.09] | 1.15 | [1.02,1.29] | 1.22 | [0.97,1.53] |
| 6-10 cigarettes | 1.10 | [1.03,1.17] | 1.39 | [1.27,1.51] | 1.38 | [1.16,1.65] |
| 11+ cigarettes | 1.23 | [1.16,1.30] | 1.66 | [1.53,1.79] | 2.15 | [1.85,2.49] |
| Alcohol: none | 0.91 | [0.82,1.01] | 0.90 | [0.78,1.04] | 1.09 | [0.84,1.42] |
| Alcohol: 1-100 gram per week | 1.00 |  | 1.00 |  | 1.00 |  |
| Alcohol: 101 or more grams per week | 1.16 | [1.10,1.22] | 1.09 | [1.01,1.17] | 1.29 | [1.13,1.48] |
| Health: Very good | 1.00 |  | 1.00 |  | 1.00 |  |
| Health: Quite good | 0.99 | [0.94,1.04] | 1.03 | [0.96,1.11] | 1.00 | [0.87,1.15] |
| Health: Neither good nor bad | 1.03 | [0.97,1.10] | 1.17 | [1.07,1.28] | 1.56 | [1.33,1.83] |
| Headache: yes, often | 1.43 | [1.27,1.60] | 1.49 | [1.27,1.74] | 1.75 | [1.31,2.33] |
| Headache: yes, sometimes | 1.18 | [1.10,1.27] | 1.18 | [1.07,1.31] | 1.32 | [1.08,1.60] |
| Headache: yes, occasionally | 1.10 | [1.03,1.17] | 1.12 | [1.02,1.23] | 1.12 | [0.93,1.34] |
| Headache: no, never | 1.00 |  | 1.00 |  | 1.00 |  |
| Stomach problems: yes, often | 1.20 | [1.07,1.35] | 1.29 | [1.11,1.50] | 1.50 | [1.14,1.98] |
| Stomach problems: yes, sometimes | 1.13 | [1.06,1.22] | 1.12 | [1.02,1.24] | 1.36 | [1.14,1.63] |
| Stomach problems: yes, occasionally | 1.04 | [0.99,1.10] | 0.99 | [0.92,1.07] | 1.02 | [0.88,1.17] |
| Stomach problems: no, never | 1.00 |  | 1.00 |  | 1.00 |  |

SD – standard deviation

**Table E.** Associations, risk ratios (95% confidence intervals), between exposures and outcomes from fully adjusted model, when using body mass index (BMI) as a measure of body size, instead of height and mass.

| Exposure | Musculoskeletal | | Cardiovascular | | Respiratory | |
| --- | --- | --- | --- | --- | --- | --- |
| BMI, per 1SD | 1.11 | [1.08,1.14] | 1.20 | [1.16,1.24] | 1.14 | [1.06,1.22] |
| Blood pressure, per 1SD | 0.98 | [0.95,1.00] | 1.11 | [1.07,1.16] | 0.98 | [0.91,1.06] |
| Pulse at rest, per 1SD | 0.93 | [0.90,0.96] | 0.95 | [0.91,0.98] | 0.98 | [0.91,1.06] |
| Hematocrit, per 1SD | 1.00 | [0.98,1.03] | 1.02 | [0.98,1.06] | 1.06 | [0.98,1.13] |
| Muscle factor, per 1SD | 1.08 | [1.05,1.11] | 1.02 | [0.98,1.06] | 0.97 | [0.90,1.05] |
| Cardiorespiratory fitness, per 50 Watt | 1.07 | [1.02,1.13] | 0.91 | [0.85,0.98] | 0.83 | [0.73,0.96] |
| Used drugs | 1.03 | [0.95,1.12] | 1.04 | [0.93,1.17] | 1.09 | [0.89,1.34] |
| Did not use drugs | 1.00 |  | 1.00 |  | 1.00 |  |
| Non smoker | 1.00 |  | 1.00 |  | 1.00 |  |
| 1-5 cigarettes | 0.96 | [0.88,1.05] | 1.13 | [0.99,1.28] | 1.27 | [0.99,1.63] |
| 6-10 cigarettes | 1.04 | [0.97,1.11] | 1.30 | [1.18,1.44] | 1.42 | [1.16,1.73] |
| 11+ cigarettes | 1.14 | [1.06,1.22] | 1.58 | [1.44,1.74] | 1.93 | [1.60,2.32] |
| Alcohol: none | 0.97 | [0.87,1.09] | 1.02 | [0.87,1.20] | 1.26 | [0.94,1.70] |
| Alcohol: 1-100 gram per week | 1.00 |  | 1.00 |  | 1.00 |  |
| Alcohol: 101 or more grams per week | 1.09 | [1.03,1.16] | 0.93 | [0.85,1.01] | 1.02 | [0.87,1.20] |
| Health: Very good | 1.00 |  | 1.00 |  | 1.00 |  |
| Health: Quite good | 0.96 | [0.91,1.02] | 0.96 | [0.89,1.04] | 0.91 | [0.78,1.07] |
| Health: Neither good nor bad | 0.94 | [0.87,1.02] | 1.01 | [0.90,1.13] | 1.16 | [0.95,1.42] |
| Headache: yes, often | 1.38 | [1.21,1.58] | 1.29 | [1.07,1.56] | 1.12 | [0.79,1.60] |
| Headache: yes, sometimes | 1.18 | [1.09,1.29] | 1.07 | [0.94,1.21] | 1.06 | [0.83,1.34] |
| Headache: yes, occasionally | 1.10 | [1.02,1.18] | 1.11 | [0.99,1.23] | 1.04 | [0.84,1.27] |
| Headache: no, never | 1.00 |  | 1.00 |  | 1.00 |  |
| Stomach problems: yes, often | 1.12 | [0.99,1.28] | 1.09 | [0.91,1.31] | 1.22 | [0.88,1.68] |
| Stomach problems: yes, sometimes | 1.05 | [0.97,1.14] | 1.04 | [0.93,1.17] | 1.13 | [0.91,1.40] |
| Stomach problems: yes, occasionally | 1.01 | [0.95,1.07] | 0.99 | [0.91,1.08] | 1.00 | [0.85,1.18] |
| Stomach problems: no, never | 1.00 |  | 1.00 |  | 1.00 |  |

SD – standard deviation

**Table F**. Associations, risk ratios (95% confidence intervals), when removing pulse at rest, cardiorespiratory fitness or both these variables from the fully adjusted model. The variables removed from the adjustment set have no estimate reported

| Exposure | Musculoskeletal | | | | | |
| --- | --- | --- | --- | --- | --- | --- |
| Mass, per 1SD | 1.12 | [1.09,1.16] | 1.13 | [1.10,1.17] | 1.14 | [1.11,1.17] |
| Height, per 1SD | 0.96 | [0.94,0.99] | 0.96 | [0.94,0.99] | 0.97 | [0.94,0.99] |
| Blood pressure, per 1SD | 0.95 | [0.93,0.98] | 0.98 | [0.95,1.00] | 0.95 | [0.93,0.97] |
| Pulse at rest, per 1SD |  |  | 0.92 | [0.90,0.95] |  |  |
| Hematocrit, per 1SD | 1.00 | [0.98,1.03] | 1.01 | [0.98,1.03] | 1.00 | [0.98,1.03] |
| Muscle factor, per 1SD | 1.08 | [1.05,1.11] | 1.08 | [1.05,1.12] | 1.09 | [1.06,1.12] |
| Cardiorespiratory fitness, per 50 Watt | 1.10 | [1.05,1.16] |  |  |  |  |
| Used drugs | 1.04 | [0.95,1.12] | 1.03 | [0.95,1.12] | 1.04 | [0.95,1.13] |
| Did not use drugs | 1.00 |  | 1.00 |  | 1.00 |  |
| Non smoker | 1.00 |  | 1.00 |  | 1.00 |  |
| 1-5 cigarettes | 0.96 | [0.88,1.05] | 0.96 | [0.88,1.05] | 0.96 | [0.88,1.04] |
| 6-10 cigarettes | 1.04 | [0.97,1.12] | 1.03 | [0.96,1.11] | 1.03 | [0.96,1.10] |
| 11+ cigarettes | 1.14 | [1.07,1.22] | 1.12 | [1.05,1.20] | 1.12 | [1.04,1.19] |
| Alcohol: none | 0.97 | [0.87,1.09] | 0.97 | [0.87,1.09] | 0.97 | [0.86,1.08] |
| Alcohol: 1-100 gram per week | 1.00 |  | 1.00 |  | 1.00 |  |
| Alcohol: 101 or more grams per week | 1.09 | [1.03,1.16] | 1.09 | [1.03,1.16] | 1.09 | [1.03,1.16] |
| Health: Very good | 1.00 |  | 1.00 |  | 1.00 |  |
| Health: Quite good | 0.95 | [0.90,1.01] | 0.95 | [0.90,1.01] | 0.94 | [0.89,1.00] |
| Health: Neither good nor bad | 0.93 | [0.86,1.01] | 0.93 | [0.86,1.01] | 0.91 | [0.84,0.99] |
| Headache: yes, often | 1.38 | [1.21,1.58] | 1.37 | [1.20,1.57] | 1.37 | [1.20,1.56] |
| Headache: yes, sometimes | 1.18 | [1.08,1.28] | 1.18 | [1.08,1.28] | 1.17 | [1.07,1.27] |
| Headache: yes, occasionally | 1.09 | [1.02,1.18] | 1.10 | [1.02,1.18] | 1.09 | [1.01,1.17] |
| Headache: no, never | 1.00 |  | 1.00 |  | 1.00 |  |
| Stomach problems: yes, often | 1.13 | [0.99,1.28] | 1.12 | [0.98,1.28] | 1.12 | [0.98,1.28] |
| Stomach problems: yes, sometimes | 1.05 | [0.97,1.14] | 1.05 | [0.96,1.14] | 1.05 | [0.96,1.14] |
| Stomach problems: yes, occasionally | 1.01 | [0.95,1.07] | 1.01 | [0.95,1.07] | 1.01 | [0.95,1.07] |
| Stomach problems: no, never | 1.00 |  | 1.00 |  | 1.00 |  |
| Exposure | Cardiovascular | | | | | |
| Mass, per 1SD | 1.22 | [1.17,1.27] | 1.21 | [1.16,1.26] | 1.21 | [1.17,1.26] |
| Height, per 1SD | 0.92 | [0.88,0.95] | 0.91 | [0.88,0.95] | 0.91 | [0.88,0.95] |
| Blood pressure, per 1SD | 1.09 | [1.06,1.14] | 1.12 | [1.07,1.16] | 1.10 | [1.06,1.14] |
| Pulse at rest, per 1SD |  |  | 0.96 | [0.92,1.00] |  |  |
| Hematocrit, per 1SD | 1.02 | [0.98,1.05] | 1.02 | [0.98,1.06] | 1.02 | [0.98,1.05] |
| Muscle factor, per 1SD | 1.01 | [0.97,1.06] | 1.00 | [0.97,1.05] | 1.01 | [0.97,1.05] |
| Cardiorespiratory fitness, per 50 Watt | 0.94 | [0.87,1.01] |  |  |  |  |
| Used drugs | 1.04 | [0.93,1.17] | 1.04 | [0.93,1.16] | 1.04 | [0.93,1.17] |
| Did not use drugs | 1.00 |  | 1.00 |  | 1.00 |  |
| Non smoker | 1.00 |  | 1.00 |  | 1.00 |  |
| 1-5 cigarettes | 1.13 | [0.99,1.28] | 1.14 | [1.00,1.29] | 1.14 | [1.00,1.29] |
| 6-10 cigarettes | 1.30 | [1.18,1.44] | 1.32 | [1.19,1.46] | 1.31 | [1.19,1.45] |
| 11+ cigarettes | 1.59 | [1.44,1.74] | 1.61 | [1.47,1.77] | 1.61 | [1.46,1.77] |
| Alcohol: none | 1.02 | [0.87,1.20] | 1.03 | [0.88,1.21] | 1.03 | [0.88,1.20] |
| Alcohol: 1-100 gram per week | 1.00 |  | 1.00 |  | 1.00 |  |
| Alcohol: 101 or more grams per week | 0.93 | [0.85,1.01] | 0.93 | [0.85,1.01] | 0.93 | [0.85,1.01] |
| Health: Very good | 1.00 |  | 1.00 |  | 1.00 |  |
| Health: Quite good | 0.95 | [0.88,1.03] | 0.96 | [0.89,1.04] | 0.96 | [0.88,1.04] |
| Health: Neither good nor bad | 1.00 | [0.90,1.12] | 1.02 | [0.91,1.14] | 1.01 | [0.91,1.13] |
| Headache: yes, often | 1.30 | [1.07,1.56] | 1.31 | [1.08,1.58] | 1.30 | [1.08,1.57] |
| Headache: yes, sometimes | 1.06 | [0.94,1.20] | 1.07 | [0.95,1.22] | 1.07 | [0.95,1.21] |
| Headache: yes, occasionally | 1.10 | [0.99,1.23] | 1.11 | [1.00,1.23] | 1.11 | [1.00,1.23] |
| Headache: no, never | 1.00 |  | 1.00 |  | 1.00 |  |
| Stomach problems: yes, often | 1.10 | [0.91,1.32] | 1.10 | [0.92,1.32] | 1.10 | [0.92,1.32] |
| Stomach problems: yes, sometimes | 1.04 | [0.93,1.17] | 1.04 | [0.93,1.17] | 1.04 | [0.93,1.17] |
| Stomach problems: yes, occasionally | 0.99 | [0.91,1.08] | 0.99 | [0.91,1.08] | 0.99 | [0.91,1.08] |
| Stomach problems: no, never | 1.00 |  | 1.00 |  | 1.00 |  |
| Exposure | Respiratory | | | | | |
| Mass, per 1SD | 1.14 | [1.05,1.23] | 1.11 | [1.03,1.21] | 1.11 | [1.03,1.21] |
| Height, per 1SD | 0.91 | [0.85,0.98] | 0.91 | [0.84,0.97] | 0.91 | [0.84,0.97] |
| Blood pressure, per 1SD | 0.98 | [0.91,1.05] | 0.99 | [0.91,1.06] | 0.99 | [0.92,1.06] |
| Pulse at rest, per 1SD |  |  | 1.01 | [0.94,1.08] |  |  |
| Hematocrit, per 1SD | 1.05 | [0.98,1.13] | 1.05 | [0.98,1.13] | 1.05 | [0.98,1.13] |
| Muscle factor, per 1SD | 0.98 | [0.90,1.06] | 0.96 | [0.89,1.04] | 0.96 | [0.89,1.04] |
| Cardiorespiratory fitness, per 50 Watt | 0.85 | [0.74,0.98] |  |  |  |  |
| Used drugs | 1.10 | [0.89,1.35] | 1.10 | [0.89,1.35] | 1.09 | [0.89,1.34] |
| Did not use drugs | 1.00 |  | 1.00 |  | 1.00 |  |
| Non smoker | 1.00 |  | 1.00 |  | 1.00 |  |
| 1-5 cigarettes | 1.27 | [0.99,1.63] | 1.28 | [1.00,1.65] | 1.28 | [1.00,1.65] |
| 6-10 cigarettes | 1.41 | [1.16,1.72] | 1.44 | [1.18,1.76] | 1.44 | [1.18,1.76] |
| 11+ cigarettes | 1.93 | [1.60,2.32] | 1.99 | [1.66,2.39] | 1.99 | [1.66,2.39] |
| Alcohol: none | 1.26 | [0.94,1.70] | 1.28 | [0.95,1.72] | 1.28 | [0.95,1.72] |
| Alcohol: 1-100 gram per week | 1.00 |  | 1.00 |  | 1.00 |  |
| Alcohol: 101 or more grams per week | 1.02 | [0.87,1.20] | 1.02 | [0.88,1.20] | 1.02 | [0.88,1.20] |
| Health: Very good | 1.00 |  | 1.00 |  | 1.00 |  |
| Health: Quite good | 0.91 | [0.78,1.07] | 0.92 | [0.79,1.08] | 0.93 | [0.79,1.08] |
| Health: Neither good nor bad | 1.16 | [0.95,1.43] | 1.19 | [0.97,1.46] | 1.19 | [0.97,1.46] |
| Headache: yes, often | 1.13 | [0.79,1.60] | 1.14 | [0.80,1.63] | 1.14 | [0.80,1.63] |
| Headache: yes, sometimes | 1.06 | [0.83,1.34] | 1.07 | [0.84,1.35] | 1.07 | [0.84,1.35] |
| Headache: yes, occasionally | 1.04 | [0.85,1.27] | 1.04 | [0.85,1.28] | 1.04 | [0.85,1.28] |
| Headache: no, never | 1.00 |  | 1.00 |  | 1.00 |  |
| Stomach problems: yes, often | 1.21 | [0.88,1.68] | 1.22 | [0.88,1.69] | 1.22 | [0.88,1.69] |
| Stomach problems: yes, sometimes | 1.13 | [0.90,1.40] | 1.13 | [0.91,1.41] | 1.13 | [0.91,1.41] |
| Stomach problems: yes, occasionally | 1.00 | [0.85,1.18] | 1.00 | [0.85,1.18] | 1.00 | [0.85,1.18] |
| Stomach problems: no, never | 1.00 |  | 1.00 |  | 1.00 |  |

SD – standard deviation

**Table G.** Associations (risk ratios with 95% confidence intervals) between exposures and specific diseases within musculoskeletal spectrum.

| Exposure | Myalgia | | Osteoarthritis | | Shoulder diseases | | Back pain | | Joint pain | |
| --- | --- | --- | --- | --- | --- | --- | --- | --- | --- | --- |
| Mass, per 1 SD | 1.15 | [1.09,1.21] | 1.19 | [1.14,1.24] | 1.11 | [1.03,1.20] | 1.07 | [1.01,1.13] | 1.10 | [1.01,1.20] |
| Height, per 1 SD | 0.97 | [0.92,1.02] | 0.93 | [0.90,0.96] | 0.82 | [0.77,0.87] | 1.01 | [0.96,1.07] | 0.94 | [0.87,1.02] |
| Blood pressure, per 1 SD | 0.95 | [0.91,1.00] | 1.00 | [0.97,1.04] | 0.95 | [0.89,1.01] | 0.96 | [0.91,1.01] | 1.01 | [0.94,1.09] |
| Pulse at rest, per 1 SD | 0.97 | [0.92,1.02] | 0.91 | [0.87,0.94] | 0.88 | [0.82,0.94] | 0.98 | [0.93,1.03] | 0.82 | [0.76,0.89] |
| Hematocrit, per 1 SD | 1.07 | [1.03,1.12] | 1.00 | [0.96,1.03] | 0.98 | [0.93,1.04] | 0.99 | [0.94,1.03] | 1.03 | [0.96,1.10] |
| Muscle strength, per 1 SD | 1.02 | [0.97,1.07] | 1.06 | [1.02,1.10] | 1.27 | [1.19,1.36] | 1.18 | [1.12,1.24] | 1.02 | [0.94,1.10] |
| Cardiorespiratory fitness, per 50 Watt | 1.00 | [0.92,1.10] | 1.23 | [1.15,1.32] | 0.93 | [0.82,1.05] | 0.97 | [0.88,1.06] | 1.12 | [0.97,1.29] |
| Non-smoker | 1.00 |  | 1.00 |  | 1.00 |  | 1.00 |  | 1.00 |  |
| 1-5 cigarettes | 0.95 | [0.82,1.11] | 1.02 | [0.91,1.13] | 1.02 | [0.83,1.25] | 1.06 | [0.90,1.25] | 0.97 | [0.77,1.23] |
| 6-10 cigarettes | 1.05 | [0.93,1.18] | 1.00 | [0.91,1.09] | 1.15 | [0.98,1.34] | 1.18 | [1.04,1.35] | 0.91 | [0.75,1.11] |
| 11+ cigarettes | 1.20 | [1.07,1.34] | 1.03 | [0.94,1.12] | 1.19 | [1.02,1.39] | 1.30 | [1.15,1.47] | 1.12 | [0.93,1.35] |
| Alcohol: none | 0.86 | [0.70,1.05] | 0.94 | [0.81,1.09] | 0.80 | [0.60,1.07] | 1.12 | [0.92,1.37] | 0.76 | [0.54,1.07] |
| Alcohol: 1-100 gram per week | 1.00 |  | 1.00 |  | 1.00 |  | 1.00 |  | 1.00 |  |
| Alcohol: 101 or more grams per week | 1.07 | [0.96,1.18] | 1.13 | [1.05,1.22] | 1.21 | [1.06,1.38] | 1.07 | [0.96,1.20] | 1.17 | [1.00,1.37] |
| Health: Very good | 1.00 |  | 1.00 |  | 1.00 |  | 1.00 |  | 1.00 |  |
| Health: Quite good | 0.98 | [0.89,1.08] | 0.93 | [0.86,1.00] | 0.96 | [0.84,1.09] | 0.98 | [0.88,1.09] | 0.85 | [0.73,0.99] |
| Health: Neither good nor bad | 0.98 | [0.85,1.12] | 0.91 | [0.82,1.02] | 0.93 | [0.77,1.12] | 0.95 | [0.82,1.11] | 0.89 | [0.71,1.11] |
| Stomach problems: yes, often | 1.20 | [0.97,1.49] | 1.09 | [0.91,1.31] | 1.29 | [0.98,1.71] | 1.14 | [0.91,1.44] | 0.94 | [0.63,1.40] |
| Stomach problems: yes, sometimes | 1.11 | [0.96,1.27] | 1.10 | [0.99,1.23] | 0.97 | [0.80,1.17] | 1.05 | [0.91,1.22] | 1.02 | [0.80,1.28] |
| Stomach problems: yes, occasionally | 1.00 | [0.90,1.11] | 1.08 | [1.00,1.17] | 0.96 | [0.84,1.10] | 0.97 | [0.87,1.09] | 1.12 | [0.95,1.31] |
| Stomach problems: no, never | 1.00 |  | 1.00 |  | 1.00 |  | 1.00 |  | 1.00 |  |
| Used drugs | 1.12 | [0.98,1.28] | 1.09 | [0.98,1.21] | 0.96 | [0.80,1.16] | 0.92 | [0.79,1.07] | 1.26 | [1.02,1.56] |
| Did not use drugs | 1.00 |  | 1.00 |  | 1.00 |  | 1.00 |  | 1.00 |  |
| Headache: yes, often | 1.53 | [1.23,1.91] | 1.23 | [1.02,1.48] | 1.65 | [1.24,2.19] | 1.74 | [1.38,2.18] | 1.17 | [0.78,1.74] |
| Headache: yes, sometimes | 1.19 | [1.03,1.38] | 1.19 | [1.06,1.33] | 1.16 | [0.95,1.41] | 1.26 | [1.07,1.47] | 1.29 | [1.02,1.63] |
| Headache: yes, occasionally | 1.07 | [0.94,1.22] | 1.17 | [1.06,1.29] | 1.04 | [0.88,1.23] | 1.10 | [0.96,1.26] | 1.12 | [0.91,1.37] |
| Headache: no, never | 1.00 |  | 1.00 |  | 1.00 |  | 1.00 |  | 1.00 |  |

SD – standard deviation

**Methods – multiple imputation.**

We created 10 imputed data sets, using multiple imputation by chained equations, through *mi impute chained* command in Stata. We included all variables included in the analysis model, and additionally an indicator for death during follow-up time. Continuous variables were imputed using linear regression, categorical variables with more than two categories with multinomial regression and binary variables with logistic regression. Prior to multiple imputation, we fitted all these models in complete cases approach to evaluate model fit. We checked the convergence of the imputation model and that imputed values were reasonable. We combined the results from imputed datasets using Rubin’s rule through *mi estimate* command in Stata. We present results based on complete cases data in the main manuscript text, and results based on multiple imputation in supplementary material. The two were almost identical.

**Table H.** Risk ratios (95%CI) for associations between exposures and the three chronic diseases from multiply imputed data. All exposures were included in one model. Additionally adjusted for parental occupation and education.

| Exposure | Musculoskeletal | | Cardiovascular | | Respiratory | |
| --- | --- | --- | --- | --- | --- | --- |
| Mass, per 1 SD | 1.12 | [1.09,1.15] | 1.23 | [1.19,1.27] | 1.13 | [1.06,1.22] |
| Height, per 1 SD | 0.97 | [0.94,0.99] | 0.92 | [0.89,0.95] | 0.90 | [0.85,0.95] |
| Blood pressure, per 1 SD | 0.97 | [0.95,1.00] | 1.10 | [1.06,1.14] | 0.98 | [0.92,1.05] |
| Pulse at rest, per 1 SD | 0.93 | [0.91,0.95] | 0.96 | [0.93,0.99] | 0.99 | [0.92,1.06] |
| Hematocrit, per 1 SD | 1.01 | [0.98,1.03] | 1.03 | [0.99,1.06] | 1.05 | [0.99,1.12] |
| Muscle strength, per 1 SD | 1.07 | [1.05,1.10] | 1.02 | [0.98,1.05] | 1.01 | [0.94,1.09] |
| Cardiorespiratory fitness, per 50 Watt | 1.06 | [1.01,1.11] | 0.90 | [0.85,0.96] | 0.84 | [0.74,0.95] |
| Non smoker | 1.00 |  | 1.00 |  | 1.00 |  |
| 1-5 cigarettes | 0.99 | [0.91,1.07] | 1.15 | [1.03,1.29] | 1.23 | [0.98,1.55] |
| 6-10 cigarettes | 1.05 | [0.99,1.12] | 1.38 | [1.26,1.51] | 1.33 | [1.11,1.60] |
| 11+ cigarettes | 1.14 | [1.07,1.21] | 1.61 | [1.48,1.76] | 1.93 | [1.63,2.29] |
| Alcohol: none | 0.96 | [0.86,1.07] | 1.04 | [0.89,1.21] | 1.33 | [1.02,1.74] |
| Alcohol: 1-100 gram per week | 1.00 |  | 1.00 |  | 1.00 |  |
| Alcohol: 101 or more grams per week | 1.09 | [1.03,1.15] | 0.94 | [0.87,1.01] | 1.03 | [0.89,1.18] |
| Health: Very good | 1.00 |  | 1.00 |  | 1.00 |  |
| Health: Quite good | 0.96 | [0.91,1.01] | 0.97 | [0.91,1.05] | 0.91 | [0.78,1.05] |
| Health: Neither good nor bad | 0.95 | [0.88,1.02] | 1.01 | [0.91,1.12] | 1.21 | [1.01,1.45] |
| Stomach problems: yes, often | 1.09 | [0.97,1.23] | 1.11 | [0.94,1.30] | 1.07 | [0.80,1.44] |
| Stomach problems: yes, sometimes | 1.07 | [0.99,1.15] | 1.02 | [0.92,1.14] | 1.14 | [0.94,1.38] |
| Stomach problems: yes, occasionally | 1.02 | [0.97,1.08] | 0.96 | [0.89,1.03] | 0.97 | [0.84,1.13] |
| Stomach problems: no, never | 1.00 |  | 1.00 |  | 1.00 |  |
| Used drugs | 1.02 | [0.95,1.10] | 1.01 | [0.91,1.12] | 1.12 | [0.93,1.35] |
| Did not use drugs | 1.00 |  | 1.00 |  | 1.00 |  |
| Headache: yes, often | 1.40 | [1.24,1.58] | 1.31 | [1.11,1.54] | 1.27 | [0.94,1.72] |
| Headache: yes, sometimes | 1.17 | [1.09,1.27] | 1.09 | [0.97,1.21] | 1.11 | [0.90,1.38] |
| Headache: yes, occasionally | 1.10 | [1.03,1.18] | 1.09 | [0.99,1.20] | 1.07 | [0.89,1.28] |
| Headache: no, never | 1.00 |  | 1.00 |  | 1.00 |  |

**Methods – normalizing cardiorespiratory fitness by body mass.**

Prompted by a reviewer comment, we performed a sensitivity analysis when using cardiorespiratory fitness normalized by body mass (W/kg) as exposure (denoted as model A below). We fitted both crude and fully adjusted models. Given that such normalized exposure is a statistical interaction (between cardiorespiratory fitness in Watt and inverse of body mass in 1/kg), we also included models with the main effect of both, as this would be in line with principles of statistical modeling (denoted as model B below) [1,2]. When only the interaction term (W/kg) is included (model A), the regression coefficient represents a combination of effect of cardiorespiratory fitness (here measured as physical work capacity in Watt) and inverse of body mass.

The exposure variables were standardized so that one unit corresponds to 1 standard deviation [SD]. This means that the unit for ratio variable in model A is based on SD of the ratio variable, in model B the units are based on SD of CRF and inverse of body mass, respectively.

1. Kronmal RA. Spurious Correlation and the Fallacy of the Ratio Standard Revisited. J R Stat Soc Ser A Stat Soc. 1993;156(3):379–92.

2. Packard GC, Boardman TJ. The use of percentages and size-specific indices to normalize physiological data for variation in body size: wasted time, wasted effort? Comp Biochem Physiol A Mol Integr Physiol. 1999 Jan 1;122(1):37–44.

**Table I.** The crude risk ratios (RR) with 95% confidence intervals (CI) for associations between cardiorespiratory fitness normalized by body mass (W/kg), with and without main effects of the interaction variables.

| Exposure and model | **Musculoskeletal** | | **Cardiovascular** | | **Respiratory** | |
| --- | --- | --- | --- | --- | --- | --- |
|  | RR | 95%CI | RR | 95%CI | RR | 95%CI |
| Model A |  |  |  |  |  |  |
| CRF/body mass (W/kg) | 0.94 | [0.92,0.96] | 0.82 | [0.79,0.84] | 0.84 | [0.79,0.90] |
| Model B |  |  |  |  |  |  |
| CRF/body mass (W/kg) | 0.97 | [0.93,1.00] | 0.95 | [0.90,1.00] | 0.91 | [0.82,1.00] |
| CRF (W) | 1.06 | [1.01,1.11] | 0.79 | [0.75,0.84] | 0.69 | [0.62,0.78] |
| Inverse of body mass (1/kg) | 0.87 | [0.85,0.89] | 0.78 | [0.76,0.81] | 0.88 | [0.82,0.94] |

**Table J.** The adjusted risk ratios (RR) with 95% confidence intervals (CI) for associations between cardiorespiratory fitness normalized by body mass (W/kg), with and without main effects of the interaction variables.

| Exposure and model | **Musculoskeletal** | | **Cardiovascular** | | **Respiratory** | |
| --- | --- | --- | --- | --- | --- | --- |
|  | RR | 95%CI | RR | 95%CI | RR | 95%CI |
| Model A |  |  |  |  |  |  |
| CRF/body mass (W/kg) | 0.96 | [0.93,0.98] | 0.86 | [0.82,0.89] | 0.88 | [0.81,0.94] |
| Model B |  |  |  |  |  |  |
| CRF/body mass (W/kg) | 0.96 | [0.92,1.00] | 0.95 | [0.89,1.01] | 0.93 | [0.83,1.04] |
| CRF (W) | 1.05 | [0.99,1.11] | 0.88 | [0.82,0.95] | 0.83 | [0.72,0.96] |
| Inverse of body mass (1/kg) | 0.88 | [0.85,0.91] | 0.79 | [0.76,0.83] | 0.86 | [0.78,0.94] |

**Code.** Stata code.

***code for statistical analysis, Poisson model with GEE

*set working directory and read in the data

cd "${lubox}\Projects\conscription_cohorts\health_young_vs_mage\work2"

use data_a_v1, clear

**describe variables

label variable outcome "Outcome, yes/no"

label variable numb "Type of outcome (MSK, CVD; respiratory)"

label variable lngdsd "Height, per 1SD"

label variable masssd "Mass, per 1SD"

label variable mfaksd "Muscle strength, per 1SD"

label variable bpresssd "Blood pressure, per 1SD"

label variable vpulsd "Pulse at rest, per 1SD"

label variable fysaconsd "Cariorespiratory fitness, per 50 Watt"

label variable smoking2 "Smoking category"

label variable alc_cat2 "Alcohol consumption, category"

label variable overallhealth2 "Overall health, category"

label variable magbesvar "Stomach problems, category"

label variable useddrugs "If used drugs, yes/no"

label variable foccu "Occupation father, category"

label variable moccu "Occupation mother, category"

label variable mor "Education, category"

label variable lopnr "Encrypted personal identification number"

xtset lopnr numb

***fully adjusted analysis model

xtpoisson outcome i.numb c.lngdsd#i.numb c.masssd#i.numb c.mfaksd#i.numb c.bpresssd#i.numb c.vpulsd#i.numb c.bevfsd#i.numb c.fysaconsd#i.numb i.smoking2#i.numb ib1.alc_cat2#i.numb i.overallhealth2#i.numb ib4.magbesvar#i.numb ib2.useddrugs#i.numb ib4.headache#i.numb i.foccu i.moccu i.mor, pa corr(unstructured)

est store multi_parsoc_pois

***export results

esttab multi_parsoc_pois using multi_pois_202410242.xls, b(%9.2f) ci tab wide nostar eform nomti noobs nonotes label replace

use data_a_v1, clear

**univariable, risk ratios

xtset lopnr numb

xtpoisson outcome i.numb c.lngdsd#i.numb c.masssd#i.numb , pa corr(unstructured)

est store unilm_pois

foreach x in mfaksd bpresssd vpulsd bevfsd fysaconsd {

xtpoisson outcome i.numb c.`x'#i.numb , pa corr(unstructured)

est store uni`x'_pois

}

foreach x in smoking2 overallhealth2 {

xtpoisson outcome i.numb i.`x'#i.numb , pa corr(unstructured)

est store uni`x'_pois

}

foreach x in magbesvar headache {

xtpoisson outcome i.numb ib4.`x'#i.numb, pa corr(unstructured)

est store uni`x'_pois

}

xtpoisson outcome i.numb ib2.useddrugs#i.numb , pa corr(unstructured)

est store uniuseddrugs_pois

xtpoisson outcome i.numb ib1.alc_cat2#i.numb , pa corr(unstructured)

est store unialc_cat2_pois

esttab unilm_pois using uni_pois_20231128.xls, b(%9.2f) ci tab wide nostar eform nomti noobs nonotes label replace

foreach x in mfaksd bpresssd vpulsd bevfsd fysaconsd smoking2 alc_cat2 useddrugs overallhealth2 magbesvar headache {

esttab uni`x'_pois using uni_pois_20231128.xls, b(%9.2f) ci tab wide nostar eform nomti noobs nonotes label append drop(1.numb 2.numb 3.numb _cons)

}

***adjusted for body size only

est clear

use data_a_v1, clear

xtset lopnr numb

foreach x in mfaksd bpresssd vpulsd bevfsd fysaconsd {

xtpoisson outcome i.numb c.`x'#i.numb c.lngdsd#i.numb c.masssd#i.numb, pa corr(unstructured)

est store uni`x'_size_pois

}

foreach x in smoking2 overallhealth2 {

xtpoisson outcome i.numb i.`x'#i.numb c.lngdsd#i.numb c.masssd#i.numb, pa corr(unstructured)

est store uni`x'_size_pois

}

foreach x in magbesvar headache {

xtpoisson outcome i.numb ib4.`x'#i.numb c.lngdsd#i.numb c.masssd#i.numb, pa corr(unstructured)

est store uni`x'_size_pois

}

xtpoisson outcome i.numb ib2.useddrugs#i.numb c.lngdsd#i.numb c.masssd#i.numb, pa corr(unstructured)

est store uniuseddrugs_size_pois

xtpoisson outcome i.numb ib1.alc_cat2#i.numb c.lngdsd#i.numb c.masssd#i.numb, pa corr(unstructured)

est store unialc_cat2_size_pois

esttab unimfaksd_size_pois using uni_size_pois_20240409.xls, b(%9.2f) ci tab wide nostar eform nomti noobs nonotes label replace

foreach x in bpresssd vpulsd bevfsd fysaconsd smoking2 alc_cat2 useddrugs overallhealth2 magbesvar headache {

esttab uni`x'_size_pois using uni_size_pois_20240409.xls, b(%9.2f) ci tab wide nostar eform nomti noobs nonotes label append drop(1.numb 2.numb 3.numb _cons)

}

***multivariable with BMI instead of mass and height

cd "${lubox}\Projects\conscription_cohorts\health_young_vs_mage\work2\revision2"

est clear

use "..\data_a_v1", clear

keep lopnr mass lngd

duplicates drop

desc, f

gen bmi=mass/(lngd/100)^2

su bmi, d

list lngd mass bmi if bmi>50 & bmi!=.

***only 8 persons above 80, we assume this was a random typing error, and thus excluding these few persons makes the data MCAR

replace bmi=. if bmi>80

su bmi, d

gen bmisd=bmi/r(sd)

save tmpbmi, replace

use "..\data_a_v1", clear

merge m:1 lopnr using tmpbmi

drop _merge

xtset lopnr numb

xtpoisson outcome i.numb c.bmisd#i.numb c.bpresssd#i.numb c.vpulsd#i.numb c.bevfsd#i.numb c.mfaksd#i.numb c.fysaconsd#i.numb ib2.useddrugs#i.numb i.smoking2#i.numb ib1.alc_cat2#i.numb i.overallhealth2#i.numb ib4.headache#i.numb ib4.magbesvar#i.numb i.foccu i.moccu i.mor, pa corr(unstructured)

est store multi_bmi_parsoc_pois

esttab multi_bmi_parsoc_pois using multi_bmi_pois_20241023.xls, b(%9.2f) ci tab wide nostar eform nomti noobs nonotes label replace

***analyses with and without adjusting for pulse at rest or cardiorespiratory fitness

est clear

use "..\data_a_v1", clear

desc, f

xtset lopnr numb

xtpoisson outcome i.numb c.lngdsd#i.numb c.masssd#i.numb c.mfaksd#i.numb c.bpresssd#i.numb c.bevfsd#i.numb c.fysaconsd#i.numb i.smoking2#i.numb ib1.alc_cat2#i.numb i.overallhealth2#i.numb ib4.magbesvar#i.numb ib2.useddrugs#i.numb ib4.headache#i.numb i.foccu i.moccu i.mor, pa corr(unstructured)

est store multi_nopulse_parsoc_pois

xtpoisson outcome i.numb c.lngdsd#i.numb c.masssd#i.numb c.mfaksd#i.numb c.bpresssd#i.numb c.vpulsd#i.numb c.bevfsd#i.numb i.smoking2#i.numb ib1.alc_cat2#i.numb i.overallhealth2#i.numb ib4.magbesvar#i.numb ib2.useddrugs#i.numb ib4.headache#i.numb i.foccu i.moccu i.mor, pa corr(unstructured)

est store multi_nofysa_parsoc_pois

xtpoisson outcome i.numb c.lngdsd#i.numb c.masssd#i.numb c.mfaksd#i.numb c.bpresssd#i.numb c.bevfsd#i.numb i.smoking2#i.numb ib1.alc_cat2#i.numb i.overallhealth2#i.numb ib4.magbesvar#i.numb ib2.useddrugs#i.numb ib4.headache#i.numb i.foccu i.moccu i.mor, pa corr(unstructured)

est store multi_nof_nop_parsoc_pois

esttab multi_nopulse_parsoc_pois multi_nofysa_parsoc_pois multi_nof_nop_parsoc_pois using multi_sens_pois_20240924.xls, b(%9.2f) ci tab wide nostar eform nomti noobs nonotes label replace

***********analyse specific MSK diseases, each disease in a separate model

cd "${lubox}\Projects\conscription_cohorts\health_young_vs_mage\work2"

use cohortdata_v3, clear

tetrachoric myalgia oa shoulder backpain jointpain

foreach x in myalgia oa shoulder backpain jointpain {

tab `x', m

}

foreach x in myalgia oa shoulder backpain jointpain {

poisson `x' lngdsd masssd mfaksd bpresssd vpulsd bevfsd fysaconsd i.smoking2 ib1.alc_cat2 i.overallhealth2 ib4.magbesvar ib2.useddrugs ib4.headache i.foccu i.moccu i.mor

est store msksub_`x'_pois

}

esttab msksub_*_pois using msksub_multi_pois_20240216.xls, b(%9.2f) ci tab wide nostar eform noobs nonotes label replace

**Fig A**. The estimates from models with different adjustment sets.

Panel A. Musculoskeletal diseases.


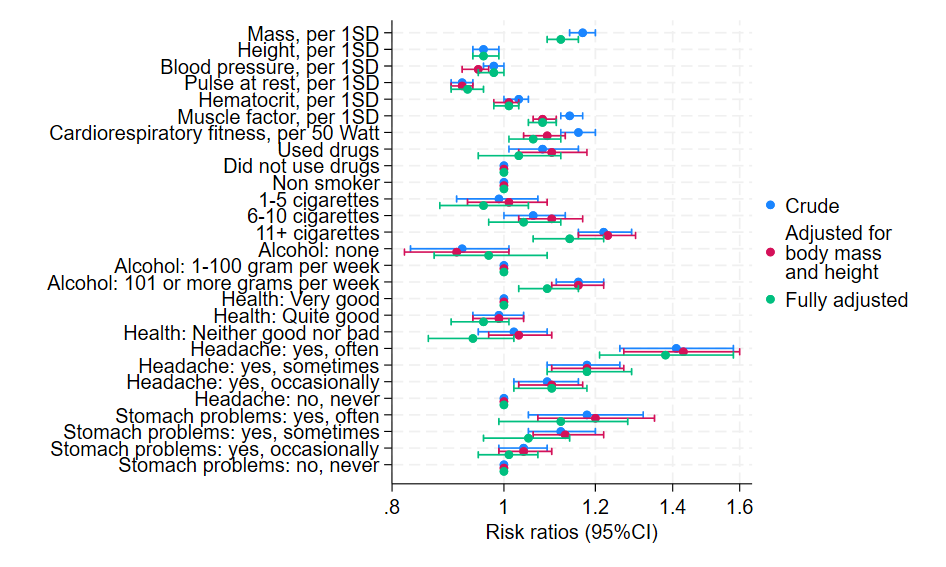


Panel B. Cardiovascular diseases.


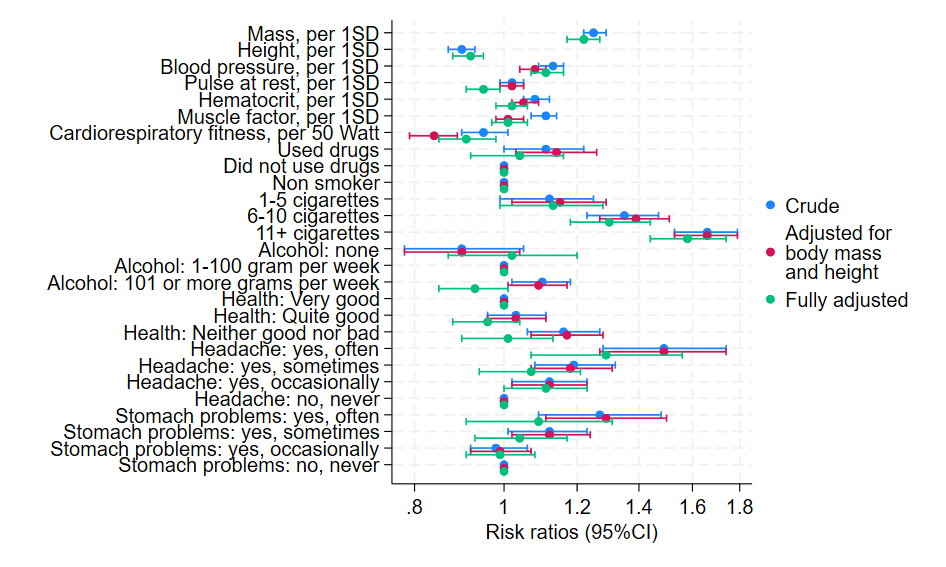


Panel C. Respiratory diseases.


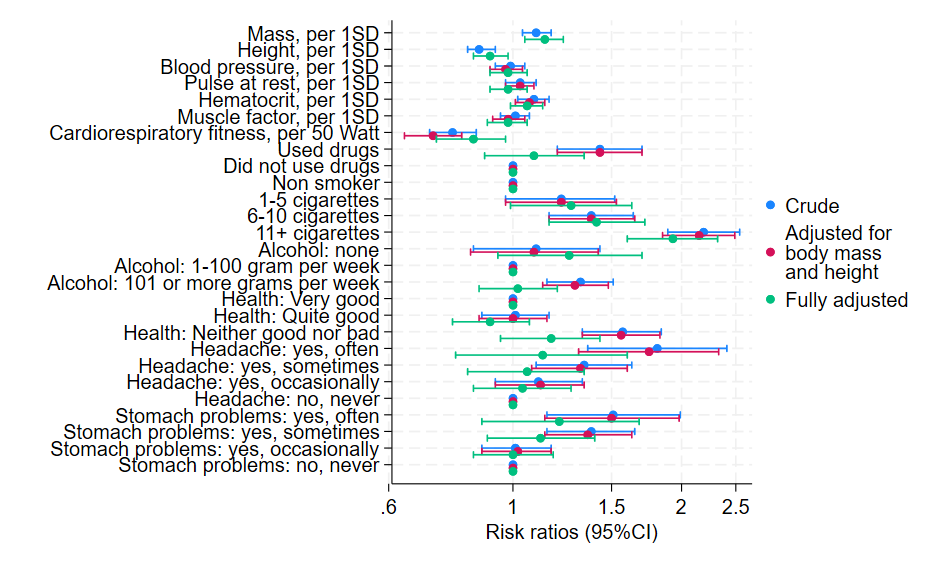

Supplement: S1 Supporting information — Table A. Correlations (Pearson’s) between the 7 continuous exposures. Table B. International Classification of Diseases (ICD)-8 diagnostic codes for identification of diseases at conscription examination. Table C. Unadjusted risk ratios (95% confidence intervals) for associations between exposure and outcomes. Body mass and height were included in 1 model. Table D. Associations, risk ratios (95% confidence intervals), between exposures and outcomes adjusted for body mass and height. Table E. Associations, risk ratios (95% confidence intervals), between exposures and outcomes from fully adjusted model, when using body mass index as a measure of body size, instead of height and mass. Table F. Associations, risk ratios (95% confidence intervals), when removing pulse at rest, cardiorespiratory fitness or both these variables from the fully adjusted model. Table G. Associations (risk ratios with 95% confidence intervals) between exposures and specific diseases within MSK spectrum. Methods–Multiple imputation. Table H. Risk ratios (95% confidence intervals) for associations between exposures and the 3 chronic diseases from multiply imputed data. All exposures were included in 1 model. Additionally adjusted for parental occupation and education. Methods–normalizing cardiorespiratory fitness by body mass. Table I. The crude risk ratios (RR) with 95% confidence intervals (CI) for associations between cardiorespiratory fitness normalized by body mass (W/kg), with and without main effects of the interaction variables. Table J. The adjusted risk ratios (RR) with 95% confidence intervals (CI) for associations between cardiorespiratory fitness normalized by body mass (W/kg), with and without main effects of the interaction variables. Code. Stata code for the main analysis model. Fig A. Risk ratio (95% confidence intervals) estimates with different sets of adjustment. Each outcome is presented in a separate panel. (DOCX) [file pmed.1004517.s002.docx]
