# Supplementary material for: Physical health in young males and risk of chronic musculoskeletal, cardiovascular, and respiratory diseases by middle age: A population-based cohort study
Source: PLoS Med. 2025 Jan 21;22(1):e1004517. doi: 10.1371/journal.pmed.1004517 (PMC11793790; doi:10.1371/journal.pmed.1004517)
Supplement: Attachment — Submitted filename: Turkiewicz_physhealth_young_point_to_point_resp_20241009.docx [file pmed.1004517.s003.docx]

We would like to thank the editorial team and the expert reviewers for their careful review of our work and insightful comments. We have responded to each of the comments, with specific actions taken. We have included quotes for the changes made and were refer to new tables and figures where relevant.

Comments from the editorial team:

The manuscript prompted a discussion about the data and the conditions listed under the musculoskeletal umbrella. We wondered if it would be possible to explore the data in more depth and with more specificity, particularly for musculoskeletal conditions. Please note that it will be important to address Reviewer #2's concerns about the analysis very carefully. We also suggest highlighting and discussing more clearly what the study adds to existing knowledge.

Authors’ response and action: We have performed reanalysis to address concerns from reviewer #2, please see specific actions taken in response to the comments from the reviewer. In brief, the results were consistent with our main findings.

We have included further information about the musculoskeletal conditions in the methods section and revised the first paragraph of discussion.

In the methods section:

“Within the musculoskeletal diseases, we selected non-inflammatory chronic pain conditions that have not well understood etiology and no disease-modifying treatment, have high prevalence in the modern society, were registered mainly as the underlying cause at the healthcare visit and are known leading causes of life years lived with disability. Thus, we included back pain, osteoarthritis, shoulder lesions, myalgia and joint pain.”

In the discussion section:

“While high body mass is a risk factor for all three studied diseases, high cardiorespiratory fitness and high muscle strength in youth are associated with increased risk of musculoskeletal disease in middle age but decreased risk of cardiovascular or respiratory diseases. ~~While our results are consistent with previous findings of risk factors in youth and future incidence of cardiovascular disease and respiratory disease, the~~ The results for musculoskeletal conditions are novel, as early risk factors of musculoskeletal conditions have not been studied previously. Further, our study is one of a kind providing estimates of associations of physical health at youth with common chronic diseases from the same population and data sources, enabling a direct comparison.”

Apart from changes made to address the comments from the editor and reviewers, we included few small changes to better comply with the RECORD checklist.
-----------------------------------------------------------
Comments from the reviewers:

Reviewer #1: Statistical review
This paper uses a longitudinal study of Swedish men to explore associations between various measures of physical health at age 18 and development of cardiovascular, respiratory and musculoskeletal diseases later in life.
I had some, mostly minor, comments on the statistical methods and reporting (although point 3 is potentially an important one to consider).

Authors’ response: Thank you for the careful review and encouraging comments.
1.      Abstract: I would recommend, if possible, providing CIs for each outcome reported in the abstract. Since a lot of associations are not reported in the abstract, it might be useful to clarify 'there were no other significant associations' (if that is the case).

Authors’ response and action: We agree. We have added specific estimates for cardiorespiratory fitness and estimates for smoking and headache and a sentence about no other large associations found. We further included a few of the most notable estimates for associations with specific musculoskeletal conditions.

New text in the abstract, methods and findings section:

“We confirmed the adverse effects of smoking, with risk ratios when comparing 11+ cigarettes per day to non-smoking of 1.14 (95% CI1.06,1.22) for musculoskeletal, 1.58 (1.44,1.74) for cardiovascular, and 1.93 (1.60,2.32) for respiratory diseases. Self-reported headache seems associated with the chronic diseases in middle age as well, with risk ratios (category often compared to never) of 1.38 (1.21,1.58) for musculoskeletal and 1.29 (1.07,1.56) for cardiovascular diseases with uncertain risk ratio of 1.13 (0.79,1.60) for respiratory diseases. No large consistent associations were found for other exposures. The most notable associations with specific musculoskeletal conditions were for cardiorespiratory fitness and osteoarthritis (risk ratio 1.23 [1.15,1.32] per 1SD) and for muscle strength and back pain (risk ratio 1.18 [1.12,1.24]) or shoulder diseases (risk ratio 1.27 [1.19,1.36]).”

2.      Page 3 - can it be clarified that the assessments were done at the beginning of conscription? If done at the end, then much less variability in fitness would presumably be expected.

Authors’ response and action: We agree. We have added this information to the methods section (added text in italic).

“We obtained data on Swedish male conscripts born in the second half of 1950 or in 1951 who underwent conscription in the years 1969 and 1970, typically at age 18 years. During this time, the data collection included measurements of physical fitness and a lifestyle questionnaire for collecting data on self-reported smoking, alcohol consumption, use of drugs and overall health, *all collected prior to the start of the military service*.”

3.      Statistical analysis: if someone died during follow-up, was the follow-up time included in the model via an offset? I am unsure of what assumption is being made if someone died of one of the causes of interest (e.g. cardiovascular disease) in terms of competing risks: I presume it is equivalent to non-informative censoring if the follow-up time is incorporated into an offset.

Authors’ response: We thank the reviewer for this important comment. We did consider this issue in the planning of the study. In this cohort, death was not very common and the overlap between the diseases was not large (e.g. 83% of the included persons had only one of the chronic diseases that were of interest). Thus, we assumed that if any of the diseases of interest was the cause of death, the person would be considered to have the disease. If someone died from other causes (without a prior disease of interest), this person would be assumed to not have the disease of interest. While we agree that this assumption could be likely violated, we consider this of lesser importance in our cohort due to relatively low age at the end of follow-up, and as a consequence, few deaths and little overlap between the diseases. We had almost only administrative censoring (apart from death and relocation from Sweden, which both were uncommon). The timing of the diagnosis in the healthcare register is not always the time of the actual incidence of the disease, especially for musculoskeletal conditions that are of our main interest, because they develop slowly over time and lead to a healthcare contact when symptoms manifest. This is why we did not include time as offset and estimated risk ratios.

Authors’ action: We have provided number of persons who died or permanently emigrated prior to having an outcome in the results section and included this issue in the discussion section:

Results:

“Among persons without any of the diseases, 1585 died while 594 emigrated from Sweden during the study time.”

Discussion:

“Another potential source of bias is censoring due to death or emigration, but in this relatively young cohort this was uncommon (~5% of the study sample), and the causes of death were typically distinct from our diseases of interest and included cancer, suicide and accidents.”

4.      Results - it might be useful to add the proportion of subjects who had the three different outcomes of interest at the follow-up.
Authors’ response: This information was already included in the second paragraph of the descriptive data section within results.

Authors’ action: No further action taken.
5.      Table 4 - I'd recommend using 'height' instead of 'length'
Authors’ response and action: We agree, we have revised as requested.

James Wason

Reviewer #2: This is an interesting analysis of the association between factors related to physical health at age ~18 years and risk of cardiovascular, respiratory and musculoskeletal disease outcomes up to age 60 years. Associations between body mass and cardiorespiratory fitness with cardiovascular and respiratory diseases were in the expected directions (body mass associated with higher risk and CRF with lower risk). However, slightly unexpectedly, CRF was assocated with higher risk of musculoskeletal disease.

Authors’ response. Thank you very much for the careful review and the encouraging comments.

However examination of the data suggests that this effect may be an artifact of the statistical analysis model. In the model both CRF (assessed as power output on a cycle ergometry test) and resting heart rate were both included in the statistical model.  Resting heart rate is a measure of fitness in its own right, so essentially the model has two different measures of CRF.  The associations of CRF (as assessed by power) and of resting heart rate on musculoskeletal disease were essentially equal but in opposite directions (0.93 for resting heart rate, 1.06 for CRF assessed by power). This suggests that the true association between CRF and muscloskeletal disease is actually close to null.  If this is the case, the whole premise and interpretation of the data will completely change.  I suggest that the authors repeat their analysis excluding resting heart rate from the model to see if the adverse association of CRF assessed by power with musculoskeletal disease persists if they do this.  They should also remove CRF assessed by power from the model, in a separate analysis to see whether the benefical association of resting heart rate with musculoskeletal disease persists.  It is likely that the entire interpretation of the data in the discussion will need to be rewritten in light of these analyses.

Authors’ response: We thank the reviewer for this insightful comment. We have now re-examined our models and results. As could be seen in the original supplementary table 2 (crude associations), cardiorespiratory fitness has similar associations with the outcomes in the unadjusted and adjusted analyses, which suggests that simultaneous adjustment for pulse at rest and CRF does not cause a change in the direction of the estimates.

In line with the reviewer’s comment, we have now also fitted three more models, with all exposures apart from pulse at rest, or apart from CRF, and without both pulse at rest and CRF. The CRF and pulse at rest are of course negatively correlated (please see also new supplementary descriptive data described in response to the reviewer#3) but the correlation is ~ -0.3 in our data (where all participants have the same sex and age, and were measured at age of 18 years, thus the correlation is naturally lower than what can be expected in the general population), and thus the model is able to estimate the contribution of both variables. All results from these analyses are presented in new S Table 6. The results were consistent, even if the specific estimates did change their values somewhat. For example, the association between cardiorespiratory fitness and MSK disease was stronger when not adjusting for pulse at rest. We include below a short table with the main estimates:

Table (for review only, the full table is provided as S Table 6). Risk ratios for muscle strength, cardiorespiratory fitness and pulse at rest from model with various adjustment sets.

|  |  | MSK | CVD | Respiratory |
| --- | --- | --- | --- | --- |
| Fully adjusted (main model) | Muscle strength (per 1SD) | 1.08 (1.05, 1.11) | 1.01 (0.97, 1.06) | 0.98 (0.90, 1.06) |
|  | Cardiorespiratory fitness (per 1SD) | 1.06 (1.01, 1.12) | 0.91 (0.85 .098) | 0.85 (0.73, .097) |
|  | Pulse at rest (per 1SD) | 0.93 (0.90, 0.96) | 0.95 (0.91, 0.99) | 0.98 (0.91, 1.06) |
| Fully adjusted, but not for pulse at rest | Muscle strength (per 1SD) | 1.08 (1.05, 1.11) | 1.01 (0.97, 1.06) | 0.98 (0.90, 1.06) |
|  | Cardiorespiratory fitness (per 1SD) | 1.10 (1.05, 1.16) | 0.94 (0.87, 1.01) | 0.85 (0.74, 0.98) |
| Fully adjusted, but not for cardiorespiratory fitness | Muscle strength (per 1SD) | 1.08 (1.05, 1.12) | 1.00 (0.97, 1.05) | 0.96 (0.89, 1.04) |
|  | Pulse at rest (per 1SD) | 0.92 (0.90, 0.95) | 0.96 (0.92, 1.00) | 1.01 (0.94, 1.08) |
| Fully adjusted, but not for cardiorespiratory fitness or pulse at rest | Muscle strength (per 1SD) | 1.09 (1.06, 1.12) | 1.01 (0.97, 1.05) | 0.96 (0.89, 1.04) |

We would like to note that in the original supplementary table 3 (each exposure analysed separately, but adjusted for body mass and height) there was an error and the estimates with MSK and CVD switched places. I.e., also in this analysis, the associations remain similar, with the risk ratio of MSK 1.09 (1.04, 1.13) per 1SD of CRF, and of CVD 0.84 (0.79, 0.89) per 1SD of CRF. We apologize for this error and have corrected it in the revised version.

Authors’ action: We have included the new results in the S Table 6. We have added description of the new analyses in the methods section.

“Prompted by comments from reviewers, we further included results adjusted for BMI (instead of body mass and height), and adjusted for pulse at rest or cardiorespiratory fitness, but not both in the same model.”

Further more minor comments include:

1) It would be helpful to include BMI rather than height and weight in the model

Authors’ response. We agree that BMI is an established variable used to describe body size, even though it has inherent statistical and interpretational problems, both from statistical perspectives (an interaction/ratio variable, ref: https://doi.org/10.2307/2983064) and from a causal inference point of view (ref: 10.1093/aje/kwp292). The main issue is that it is a ratio variable created by dividing two body size measures. In a statistical sense, it is an interaction, while it is typically analyzed without including the main terms for the two variables in the model. We fully agree that for completeness and comparisons with other studies, it is important to include results from models adjusted for BMI instead of body mass and height.

Authors’ action: We have included a new S Table 5 (also in line with a comment from reviewer #3) when we used BMI instead of body mass and height. The results were essentially the same. We clarify in the methods section, why we did not use BMI in the main analysis.

“We avoided using body mass index in the main model, due to its complicated statistical (a ratio variable) and epidemiological interpretations [20,21], but we used it in a sensitivity analysis and to enable comparisons with other studies.”

2) It would be helpful to express CRF in W/kg, rather than in W as this relates more to VO2max measured in ml/kg/min

Authors’ response. We agree that it could be useful for the reader to see CRF also expressed in relation to body mass, we have included this information in the descriptive Table 3. Given that normalizing CRF to body mass would lead to inappropriate statistical analysis (please see: https://doi.org/10.2307/2983064), we politely suggest not changing the analysis model.

Authors’ action: We have added mean values in Watt/kg in the Table 3.
3) It would be helpful to see the relationship between muscular strength and the outcomes in a model which does not also include CRF or resting heart rate.

Authors’ response. We agree. Please see our answer to your main comment above.
4) In table 4 and in places in the text, replace 'length' with 'height' 
Authors’ response and action. We agree and have revised accordingly.

Reviewer #3: This is a well-conducted and well-written study which produces some interesting results, particularly on the contrasting associations of early-life fitness and strength on cardiovascular versus musculoskeletal conditions in middle age. I very much hope to see it published soon. There are a couple of statistical issues where I would like to see sensitivity analyses in the supplement to reassure me (and the reader of the published article) that some non-obvious analytical choices have not greatly influenced the findings. The first is the choice to analyse linearly-adjusted body mass rather than BMI (see major issue below) and the second is the presentation of mutually-adjusted results for all exposures (see minor issue Results, para 4 below). Other than that, I have listed in the minor issues every place I could see where I thought the clarity of the manuscript could be improved. These are numerous but mostly very trivial and easily remedied.

Authors’ response. Thank you very much for the careful review and the encouraging comments.
Major issues:
BMI is commonly used to measure adiposity, rather than adjusting analyses of body mass for the person's height as you have done. Linear adjustment for height may not be the best approach since we expect body mass to increase more than linearly with height from simple geometry. Adjusting for height rather than using a ratio with a standard exponent also makes the assumption (rightly or wrongly) that the true medically-relevant measure of mass-for-height is independent of height. If you believe you have a good justification for your approach, rather than using BMI, it should be given in the manuscript. Furthermore, unless you have a really convincing argument against using BMI at all, I would like to see a sensitivity analysis in the supplement showing (hopefully) that analysis of BMI produces much the same conclusions as your analysis of body mass adjusted linearly for height.

Authors’ response. We agree that BMI is an established variable used to describe body size, even though it has inherent statistical and interpretational problems, both from statistical perspectives (an interaction/ratio variable, ref: https://doi.org/10.2307/2983064) and from a causal inference point of view (ref: 10.1093/aje/kwp292). The main issue is that it is a ratio variable created by dividing two body size measures. In a statistical sense, it is an interaction, while it is typically analyzed without including the main terms for the two variables in the model. We fully agree that for completeness and comparisons with other studies, it is important to include results from models adjusted for BMI instead of body mass and height.

Authors’ action: We have included a new S Table 5 (also in line with a comment from reviewer #3) when we used BMI instead of body mass and height. The results were essentially the same. We clarify in the methods section, why we did not use BMI in the main analysis.

“We avoided using body mass index in the main model, due to its complicated statistical (a ratio variable) and epidemiological interpretations [20,21], but we used it in a sensitivity analysis and to enable comparisons with other studies.”

Minor issues:

Authors’ response: Thank you for the careful review. To avoid too much spurious text in our responses, for the points below that only required minor changes, we included an “authors’ response and action” line that applies to all comments above this line and below a previous “authors’ response”.

Abstract: Add "people" to "middle-aged and elderly people".
Abstract: In the results, rephrase the reporting of the body mass results to the style used for other exposures, so that the direction of the association is made explicit.
Abstract: The units of the risk ratios (per SD) are missing when they are reported for body mass.
Intro, para 1: In the first sentence, the words "years lived with" are unnecessarily specific and I would delete them.
Intro, para 1: Should be "middle-aged persons", not "middle-age persons"
Intro, para 3: "which was mandatory", not "that was mandatory"
Intro, para 3: In "they offer a unique data source for studying…", "they" is ill-defined. Replace it with "these registers" if that's what you meant. Later in the sentence, there's also a missing "a" in "as a risk factor" .
Intro, para 3: I don't think "OA" has been defined or expanded yet. I don't think the abbreviation is used again, so I would just write it out in full.
Methods, para 1: "who underwent conscription", not "that underwent conscription". It's probably not necessary to specify "the year" in "the year 1950" (etc).
Methods, para 1: I would write "during this time", not "during this year" to describe examinations in 1969 and 1970. If it's only part of each year, adding up to a one year period, please specify this.
Methods, para 1: There's a missing "in" in "The total number of conscripts in these years…"

Authors’ response and action. We have revised as requested.

Methods, para 1: You report later that you also excluded people who had an outcome in 1987 or who died before this time - this would be best reported here, along with any other exclusions.

Authors’ response and action. We agree. We have moved the information that only persons alive in 1987 were included to the Methods section, first paragraph (study cohort). We politely suggest not changing the location of the description of exclusion of persons with diseases of interest at the conscription examination, as we think that the reader needs to first be given information about the outcomes of interest.

Table 1: Be a bit more informative in the description of Hematocrit - percentage of what?

Authors’ response and action. We have updated the table 1 with definition of hematocrit (volume percentage of red blood cells in blood).
Table 1: For Cardiorespiratory fitness - how was the power output measured?

Authors’ response and action. We have added a brief description in the methods section (just below the table 1), we refer the reader to reference nr 17 for further details.

“Cardiovascular fitness (physical work capacity) was tested by means of the maximal work test, where the tested person is required to work, until reaching the point of exhaustion, on a bicycle ergometer with an invariable, heavy load. The load was chosen individually with respect to constitution and prior history of physical activity.”

Table 1: At the end of the alcohol use section there's a comma which should be a full stop.
Methods, para 7: State here that you used a log link.
Methods, para 7: The description of the data structure is a bit ambiguous. Perhaps writing "one data row" instead of "one row with data" could help. At present, it could be interpreted as saying that there was one row per person, containing data for all three outcomes (which I don't think was the case).
Methods, para 8: When you write "length" here, do you mean "height"? "Length" is also used in some tables and supplementary tables. I suggest using "height" throughout to avoid confusion.

Authors’ response and action. We have revised as requested.
Methods, para 8: When you write "Finally", it could be taken to mean that adding the interaction between exposures and outcome type was  an additional adjustment set separate from the three already described. I think you must in fact have done it in every model, given the combination of data for all three outcomes and the need to report separate RR. Please can you clarify this? Also, were the adjustment variables in the third adjustment set (parents' occupation, etc) also allowed to interact with outcome type? This would give a closer analogy to the results you'd get from separate models for each outcome because the associations with these adjustment variable might well be different for different outcomes but if you don't interact them you're forcing, for example, father's occupation to have the same association with all three outcomes. Please clarify and justify your choice.

Authors’ response and action. Thank you. We rephrased the “finally” to “In each model”. The reviewer is correct that we assumed the same relationship of parental education and occupation with all three outcomes. This may be a simplification, but as these were not our main exposures of interest, we considered that this would allow sufficient confounding control, while keeping the total number of model parameters lower. From the literature, the association of the socioeconomic factors with all three disease groups goes in the same direction (i.e. the higher the socioeconomic status, the lower incidence of the diseases). Given the already rich supplementary material we politely suggest not including another set of sensitivity analyses. We do clarify the modeling choice in the methods section:

“No interaction of outcome type with confounders was included, as the socioeconomic factors generally have the same direction of associations with all three studied disease groups (ref: PMID: 31733042).”
Methods, para 9: It's clear from the results tables that categorical variables were modelled as categories relative to a reference category, but it would be helpful to confirm that here (i.e. that you didn't dichotomise them or treat them as continuous)

Authors’ response: Yes, the reviewer is correct. We have further clarified this in the table 1. It is also visible in the results table, where the estimates are provided for each category.
Results, para 3: Please state that these incidences are for the period 1987-2010, rather than just until 2010.
Table 3: Please present the exposures in the same order in all Tables & Figures. This just makes it easier to read.
Table 3: I think those means with only two significant figures (i.e. body mass, pulse and hematocrit) and their corresponding SD should be reported to one decimal place for a little more precison.
Table 3: I think you report the mean and SD of hematocrit, in units of % (RBC) but "(%)" is written in brackets like the % for categorical variables. Other units for continuous variables are given after a comma, so shouldn't this be the case for hematocrit? Also, pulse presumably has units of minute^-1 (i.e. per minute) - this should be added to the table.
Results, para 4: I find it confusing and incomplete to report the 95% CI without the estimate and without saying that they are 95% CI. Rather than "5-11%", please write 8% (95% CI: 5%, 11%) instead (and similarly for the other results reported in this way).

Authors’ response and action. We have revised as requested.
Results, para 4: You refer to the unadjusted / partially adjusted results in Online supplementary tables 2 and 3, but you only say that they also varied in those tables. I think the reader will want to know whether the alternative adjustments made a big or small difference to the estimates - this helps us to know whether they are likely to be confounding/mediating each other. Eyeballing the supplementary tables, it looks to me as if results are broadly similar whichever adjustment set you use, but it would be much easier to see if you could add plots, similar to Figure 2 but with the different adjustment sets in place of the different musculoskeletal conditions, to the supplement so that the reader can quickly compare the unadjusted, mass&height-adjusted and fully-adjusted estimates. You should also briefly report on how influential the choice of adjustment was, in the main manuscript Results section.

Authors’ response and action: We agree. We have included a new S Figure 1 where we plot the estimates from the unadjusted and adjusted models next to each other. We also refer to this in the results text (*Associations with the musculoskeletal, respiratory, and cardiovascular diseases)*:

“The magnitude of change in the estimates due to adjustments can be seen in S Figure 1”
Results, para 4: The supplementary tables themselves are titled "supplementary table X" - unless you are following a particularly misguided journal style, it would be clearer if they could be referred to in the same way in the text.

Authors’ response and action: Thank you, all supplementary tables and figure were renamed with a prefix S.
Table 4: Because the Exposure descriptions wrap lines, it's sometimes hard to see which numbers correspond to which Exposures. Indenting wrapped lines might help, and/or including risk ratios of 1 (no CI) for the reference categories (as you do in the supplement).

Authors’ response: The tables will most probably have different aspect ratio when published. We have included a line for each reference category with a risk ratio 1 and made the column with exposure names wider, to avoid wrapped text.
Results, para 5: These risk ratios have been considerably rounded. Please either report them to the same precision they are in Table 4 ("1.93 times higher" and "58% higher") or precede them with "about" or "almost".

Authors’ response and action. We have specified 58% specifically, added a tilda in the “~2 times higher” statement and revised para 6 where we now provide the actual estimates with 95% confidence intervals.
Figure 1: Why is height excluded, when it is included in all other results? Please either include it or justify the omission.

Authors’ response: The reason is pragmatic. Having 6 panels in the figure is more esthetically appealing than 7 (and easier to make readable). We considered height as “the least interesting” exposure, as height in adulthood is not a modifiable risk factor. We politely suggest keeping the figure as is, unless the editorial office will request including the seventh panel.
Figure 1: In the figure text, please add the word "fitted" at the beginning to make it clear that these are fitted values (rather than observed values making a remarkably good linear approximation). I think the phrase "incidence proportion" also needs a bit of explanation to help the figure stand alone - it's the proportion of people developing the condition during the period 1987-2010, isn't it?
Figure 1: Again in the figure text, it took me a while to understand the last sentence. I think it's the "increments" that are ambiguous. Perhaps refer to tick marks and/or points instead? Or you could even use different symbols - something like "hollow symbols indicate the mean of each exposure, with filled symbols at increments of one standard deviation".

Figure 1: Colour-blind people may struggle to read this graph - could you perhaps use different line types or different symbol shapes in addition to  the colours?

Authors’ response and action. We have revised figure text to make it simpler but informative. We have changed the colors in the figure, but did not use different symbols. We hope that the reviewer will find it acceptable.
Figure 2: Please use consistent names for the exposures and present them in a consistent order. There's a typo in "fittness".
Figure 2: Although it's theoretically possible to guess the colour code from the ordering, giving the different musculoskeletal conditions different symbol shapes (on the graph and in the legend) might be helpful for people with colour blindness.
Discussion, para 3: It should be "…that manifest as or lead to…"
Discussion, para 3: The final sentence ("Evaluating the effectiveness…") is very long and difficult to follow. Please can you re-phrase it? It might be best broken into two sentences.
Discussion, para 4: Should be  "…earlier reports of a linear increase…" and "…even in the normal range"
Discussion, para 4: Please give the values of BMI which define the normal range.
Discussion, para 5: "less specific" would be better than the double negative "more unspecific"

Authors’ response and action: We have revised as requested.
Discussion, para 7: The reference to adjustment in the limitations section emphasises that it would be useful to have a brief report in the main manuscript and easy-to-read report in the supplement, to show whether the different adjustment sets considered here affected estimates.

Authors’ response and action: We have included a new S Figure 1.
Discussion, para 7: Could you perhaps include a correlation matrix of the exposures in the supplement, to further inform consideration of the relationships between them ?

Authors’ response and action: We agree. We have included a correlation table (S table 2) to describe the relationships between the continuous exposures. We also included a figure showing estimates from models with varying adjustment sets (S Figure 1).
Discussion, para 7: State why you might want to adjust for early life factors (presumably, as potential confounders).

Authors’ response. We have revised as requested.
Discussion, para 7: Another limitation to mention here is that you excluded people with one outcome condition in 1987 from the analysis of the others. You report that most people with one or more conditions had only one, but there's still potential for this to have biased estimates somewhat towards the null. You could check for this by doing single outcome analyses with and without the people who were excluded for having other outcomes.

Authors’ response: We agree but given that the proportion persons excluded was so low, we do not expect this selection to result in bias. Also, most of the studied diseases have incidence in middle age, and so it could be seen as a strength to start with a cohort free of all three disease groups (at least as assessed at the conscription examination). We politely suggest not including another sensitivity analysis.
Discussion, para 7: In terms of generalisation, the male-only data must also be a limitation.

Authors’ response. Yes, we now highlight this limitation in the “Author summary” section and in the discussion section.
Supplementary Table 3: Should be "…adjusted for body mass and height".

Authors’ response. We have revised as requested.

Any attachments provided with reviews can be seen via the following link:
--------------------------------------------------------- ---
General editorial requests:
(Note: not all will apply to your paper, but please check each item carefully)

*       We ask every co-author listed on the manuscript to fill in a contributing author statement, making sure to declare all competing interests. If any of the co-authors have not filled in the statement, we will remind them to do so when the paper is revised. If all statements are not completed in a timely fashion this could hold up the re-review process. If new competing interests are declared later in the revision process, this may also hold up the submission. Should there be a problem getting one of your co-authors to fill in a statement we will be in contact. Please do not add or remove authors without first discussing this with the handling editor. You can see our competing interests policy here: <http://journals.plos.org/plosmedicine/s/competing-interests>.

Authors’ response: All conflicts of interest are listed in the corresponding manuscript section. The authors contributions are stated in “Statements and Declarations” section.

*       Please upload any figures associated with your paper as individual TIF or EPS files with 300dpi resolution at resubmission; please read our figure guidelines for more information on our requirements: <http://journals.plos.org/plosmedicine/s/figures>. While revising your submission, please upload your figure files to the PACE digital diagnostic tool, <https://pacev2.apexcovantage.com/>. PACE helps ensure that figures meet PLOS requirements. To use PACE, you must first register as a user. Then, login and navigate to the UPLOAD tab, where you will find detailed instructions on how to use the tool. If you encounter any issues or have any questions when using PACE, please email us at [PLOSMedicine@plos.org](mailto:PLOSMedicine@plos.org).

Authors’ response: Done.

*       Please ensure that the paper adheres to the PLOS Data Availability Policy (see <http://journals.plos.org/plosmedicine/s/data-availability)>, which requires that all data underlying the study's findings be provided in a repository or as Supporting Information. For data residing with a third party, authors are required to provide instructions with contact information (web or email address) for obtaining the data. Please note that a study author cannot be the contact person for the data. PLOS journals do not allow statements supported by "data not shown" or "unpublished results." For such statements, authors must provide supporting data or cite public sources that include it.

Authors’ response: We have included a “Data availability statement”.

*       We expect all researchers with submissions to PLOS in which author-generated code underpins the findings in the manuscript to make all author-generated code available without restrictions upon publication of the work. In cases where code is central to the manuscript, we may require the code to be made available as a condition of publication. Authors are responsible for ensuring that the code is reusable and well documented. Please make any custom code available, either as part of your data deposition or as a supplementary file. Please add a sentence to your data availability statement regarding any code used in the study, e.g. "The code used in the analysis is available from Github [URL] and archived in Zenodo [DOI link]" Please review our guidelines at <https://journals.plos.org/plosmedicine/s/materials-software-and-code-sharing> and ensure that your code is shared in a way that follows best practice and facilitates reproducibility and reuse. Because Github depositions can
be readily changed or deleted, we encourage you to make a permanent DOI'd copy (e.g. in Zenodo) and provide the URL.

Authors’ response: Given that the data used in the project are protected by Swedish law and can not be shared, most of the code used to generate the results is related to data management that can not be reproduced without access to the data. Thus, we have included the code for the statistical analysis model as supplementary material.

*       DATA AVAILABILITY: If the data are not freely available, please describe briefly the ethical, legal, or contractual restriction that prevents you from sharing it. Please also include an appropriate contact (web or email address) for inquiries (again, this cannot be a study author).

Authors’ response: We have included a “Data availability statement”.

FORMATTING - GENERAL
*       Abstract: Please structure your abstract using the PLOS Medicine headings (Background, Methods and Findings, Conclusions). Please combine the Methods and Findings sections into one section.

Authors’ response: Done.

*       At this stage, we ask that you include a short, non-technical Author Summary of your research to make findings accessible to a wide audience that includes both scientists and non-scientists. The Author Summary should immediately follow the Abstract in your revised manuscript. This text is subject to editorial change and should be distinct from the scientific abstract. Ideally each sub-heading should contain 2-3 single sentence, concise bullet points containing the most salient points from your study. In the final bullet point of 'What Do These Findings Mean?', please include the main limitations of the study in non-technical language. Please see our author guidelines for more information: <https://journals.plos.org/plosmedicine/s/revising-your-manuscript#loc-author-summary>.

Authors’ response: We have included a non-technical Author Summary.

*       Please express the main results with 95% CIs as well as p values. When reporting p values please report as p<0.001 and where higher as the exact p value p=0.002, for example. Throughout, suggest reporting statistical information as follows to improve clarity for the reader "22% (95% CI [13%,28%]; p</=)". Please be sure to define all numerical values at first use.

Authors’ response: Given the known limitations of p-values and the recommendations from the America Statistical Associations (refs: https://doi.org/10.1080/00031305.2016.1154108), we politely suggest not including p-values in the results text, we do report all estimates with 95% confidence intervals.

*       Please include page numbers and line numbers in the manuscript file. Use continuous line numbers (do not restart the numbering on each page).

Authors’ response: Done.

*       Please cite the reference numbers in square brackets. Citations should precede punctuation.

Authors’ response: Done.

FIGURES AND TABLES
*       Please provide titles and legends for all figures and tables (including those in Supporting Information files).
Authors’ response: Done.
*       Please define all abbreviations used in each figure/table (including those in Supporting Information files).

Authors’ response: Done.

*       Please consider avoiding the use of red and green in order to make your figure more accessible to those with color blindness.

Authors’ response: Done.

SUPPLEMENTARY MATERIAL
*       Please note that supplementary material will be posted as supplied by the authors. Therefore, please amend it according to the relevant comments outlined here.

*       Please cite your Supporting Information as outlined here: <https://journals.plos.org/plosmedicine/s/supporting-information>

Authors’ response: Done.

REFERENCES
*       PLOS uses the numbered citation (citation-sequence) method and first six authors, et al.

Authors’ response: Done.

*       Please ensure that journal name abbreviations match those found in the National Center for Biotechnology Information (NCBI) databases (<http://www.ncbi.nlm.nih.gov/nlmcatalog/journals>), and are appropriately formatted and capitalised.

Authors’ response: Done.

*       Where website addresses are cited, please include the complete URL and specify the date of access (e.g. [accessed: 12/06/2024]).

Authors’ response: Done.

*       Please also see <https://journals.plos.org/plosmedicine/s/submission-guidelines#loc-references> for further details on reference formatting.

STUDY TYPE-SPECIFIC REQUESTS

*       Abstract: Please include the study design, population and setting, number of participants, years during which the study took place (enrollment and follow up), length of follow up, and main outcome measures.

Authors’ response: Done.

*       Please ensure that the study is reported according to the RECORD guideline (available from [[https://www.record-statement.org)]https://www.record-statement.org](https://www.record-statement.org)]https:/www.record-statement.org)) and include the completed checklist as Supporting Information. Please add the following statement, or similar, to the Methods: "This study is reported as per the Reporting of Studies Conducted using Observational Routinely-Collected Data (RECORD) guideline (S1 Checklist)." When completing the checklist, please use section and paragraph numbers, rather than page numbers.

Authors’ response: Done.

*       For all observational studies, in the manuscript text, please indicate: (1) the specific hypotheses you intended to test, (2) the analytical methods by which you planned to test them, (3) the analyses you actually performed, and (4) when reported analyses differ from those that were planned, transparent explanations for differences that affect the reliability of the study's results. If a reported analysis was performed based on an interesting but unanticipated pattern in the data, please be clear that the analysis was data driven.

Authors’ response: Done.

*       Please state in the Methods section whether the study had a prospective protocol or analysis plan. If a prospective analysis plan (from your funding proposal, IRB or other ethics committee submission, study protocol, or other planning document written before analyzing the data) was used in designing the study, please include the relevant document(s) with your revised manuscript as a Supporting Information file to be published alongside your study and cite it in the Methods section. A legend for this file should be included at the end of your manuscript. If no such document exists, please make sure that the Methods section transparently describes when analyses were planned, and when/why any data-driven changes to analyses took place. Changes in the analysis, including those made in response to peer review comments, should be identified as such in the Methods section of the paper, with rationale.

Authors’ response: Methods section describes what was done.
