# Supplementary material for: Physical health in young males and risk of chronic musculoskeletal, cardiovascular, and respiratory diseases by middle age: A population-based cohort study
Source: PLoS Med. 2025 Jan 21;22(1):e1004517. doi: 10.1371/journal.pmed.1004517 (PMC11793790; doi:10.1371/journal.pmed.1004517)
Supplement: Attachment — Submitted filename: Point_to_point_response_Turkiewicz_20241030.docx [file pmed.1004517.s004.docx]

We would like to sincerely thank all the reviewers for their careful review, relevant comments and interest in our work. We understand that review work requires a lot of time and effort, and we are grateful that the reviewers wished to put this time into improving our work. We provide a point by point response below. We hope that the reviewers will find the revised work acceptable.

Reviewer #1: Thank you to the authors for addressing my previous comments (and those of the other reviewers). I have no further issues to raise.

Authors’ response: Thank you for the review and the positive feedback.

Reviewer #2: Thank you for making these changes which have improved the manuscript.  I appreciate the effort in this comprehensive revision, which has addressed most of my concerns.  I would still like you to repeat the analysis expressing CRF in terms of W/kg (both with and without resting heart rate in the model). VO2max or maxiumum power expressed per kg body mass is the classic way of expressing CRF as this is the metric which is most strongly associated with indices of physical function/performance (e.g. 10K running time). A 50kg person with maximum power of 150W and a 100kg person with maximum power of 300W would have similar running performance. So W/kg is the appropriate unit for CRF here (and the effects on performance are the same whether this goes up via increasing W or decreasing kg). The fact that CRF expressed in W is not the most appropriate metric is clear from the relatively strong correlation with body mass (r = 0.39). This is counterintuitive for a measure
of CRF - the fastest people in endurance events tend to be the lightest, not the heaviest people. Expressing CRF in W/kg may also change the strength of association with resting heart rate.  Could you please undertake these analyses in a revised version?

Authors’ response: Thank you for the careful review and the positive feedback. We understand that using ratio variables (for example W/kg) is common. Despite this being a common practice, it is typically misleading. It has been described by many authors. One excellent paper with very informative examples is from Kronmal R in the Journal of the Royal Statistical Society, Statistics in Society, Series A (https://doi.org/10.2307/2983064), another informative paper is from Packard et al. (https://doi.org/10.1016/S1095-6433(98)10170-8).

Citing after Kronmal R:

“Using the ratio of stroke volume of the heart divided by body weight, Tanner (1949) described a disease of thin people caused by 'statistical artifact'. He warned of the danger of attempting to adjust for a body size variable by dividing the variable of interest by it.”

We interpret the reviewer’s comment, as if the reviewer is concerned that the body mass has not been accounted for in our analysis. However, when adjusting for body mass, the estimate for CRF in Watt is the average association as if all persons had the same body mass (in other words, when removing the effect of body mass). Importantly, dividing CRF by body mass *does not* remove confounding from body mass. Citing after Kronmal R:

“Section 4 demonstrates that the use of a ratio as an independent variable provides an inadequate adjustment for each of the variables appearing in the ratio and thus can lead to incorrect inference about the effects of the independent variables on the dependent variable.”

In other words, using a ratio variable where one divides an exposure (here CRF in Watt) by body mass would be erroneous. Nevertheless, we have fitted a series of such models to exemplify the issues and how they affect the estimates. For simplicity, we did this in models with body mass (or inverse of it) and CRF, with and without including pulse at rest and ignoring the other variables (as they had little influence on the estimates, and are not needed for between-model comparisons).

The CRF/mass variable (W/kg) is an interaction. Given that this is an interaction variable, basic principles for statistical modeling mean that also the components of the variables (main effects) need to be included, i.e. inverse of body mass (1/kg) and CRF (W). This would mean that we expect to have a linear (vs log outcome) association with the inverse of the body mass. This is unlikely, as what we expect (and what we did check in our data), is a linear association with body mass; not the inverse of it. This is why, when adjusting for body mass, one does not typically use the inverse of body mass. Even when various authors use BMI, the mass is in the *numerator*, i.e. they are not using the inverse of body mass.

Thus, model A below includes only the interaction between CRF and inverse of body mass (W/kg), with or without pulse at rest. We note that this model is invalid due to two reasons: it does not include the main effects of the variables included in the interaction, and it does not remove confounding from body mass.

Model B is like model A above but includes also the main effects of CRF and the inverse of body mass. It is a better model than model A but could still be misspecified by a potentially non-linear association with the inverse of body mass.

Model C includes CRF, body mass (not inverse of it), but with an interaction between CRF and body mass.

All exposure variables were standardized so that 1 unit corresponds to 1 SD. This means that the unit for ratio variable in model A is based on SD of the ratio variable, in model B the units are based on SD of CRF and inverse of body mass, respectively, while in model C the units are based on SD of CRF and body mass.

Table for review. The risk ratio estimates (95%CIs) from Poisson models described above.

|  | MSK | | CVD | | Respiratory | |
| --- | --- | --- | --- | --- | --- | --- |
| **Model A without pulse at rest** |  |  |  |  |  |  |
| CRF/body mass (W/kg) | 0.94 | [0.92,0.96] | 0.82 | [0.79,0.84] | 0.84 | [0.79,0.90] |
| **Model A with pulse at rest** |  |  |  |  |  |  |
| CRF/body mass (W/kg) | 0.92 | [0.90,0.94] | 0.81 | [0.79,0.84] | 0.84 | [0.79,0.90] |
| Pulse at rest | 0.90 | [0.88,0.92] | 0.98 | [0.95,1.01] | 0.99 | [0.94,1.06] |
| **Model B without pulse at rest** |  |  |  |  |  |  |
| CRF (W) | 1.06 | [1.01,1.11] | 0.79 | [0.75,0.84] | 0.69 | [0.62,0.78] |
| Inverse of body mass (1/kg) | 0.87 | [0.85,0.89] | 0.78 | [0.76,0.81] | 0.88 | [0.82,0.94] |
| Interaction between CRF and inverse of body mass | 0.97 | [0.93,1.00] | 0.95 | [0.90,1.00] | 0.91 | [0.82,1.00] |
| **Model B with pulse at rest** |  |  |  |  |  |  |
| CRF (W) | 1.01 | [0.97,1.06] | 0.79 | [0.74,0.84] | 0.68 | [0.61,0.77] |
| Inverse of body mass (1/kg) | 0.86 | [0.84,0.89] | 0.78 | [0.76,0.81] | 0.88 | [0.82,0.94] |
| Interaction between CRF and inverse of body mass | 0.97 | [0.93,1.01] | 0.95 | [0.90,1.00] | 0.91 | [0.83,1.00] |
| Pulse at rest | 0.92 | [0.90,0.95] | 0.99 | [0.96,1.03] | 0.98 | [0.92,1.04] |
| **Model C without pulse at rest** |  |  |  |  |  |  |
| CRF (W) | 1.08 | [1.03,1.12] | 0.82 | [0.77,0.87] | 0.70 | [0.63,0.79] |
| Body mass (kg) | 1.13 | [1.11,1.16] | 1.25 | [1.21,1.28] | 1.13 | [1.06,1.20] |
| Interaction between CRF and body mass | 1.02 | [0.98,1.06] | 1.03 | [0.98,1.08] | 1.09 | [0.99,1.20] |
| **Model C with pulse at rest** |  |  |  |  |  |  |
| CRF (W) | 1.03 | [0.98,1.07] | 0.81 | [0.76,0.86] | 0.69 | [0.61,0.78] |
| Body mass (kg) | 1.14 | [1.12,1.17] | 1.25 | [1.21,1.29] | 1.13 | [1.06,1.20] |
| Interaction between CRF and body mass | 1.01 | [0.98,1.05] | 1.03 | [0.98,1.08] | 1.08 | [0.99,1.19] |
| Pulse at rest | 0.92 | [0.90,0.95] | 0.99 | [0.96,1.02] | 0.98 | [0.92,1.04] |

We note that both model B and model C provide no evidence of interactions, although model B could indicate a small interaction, it uses the inverse of body mass and thus the assumption of linearity may be violated.

Both models B and C suggest that higher body mass (i.e. lower inverse of body mass) is associated with higher risks of all three diseases (ignoring the interaction term, a model without interaction should be fitted). The estimate from model A lacks proper interpretation. Both models B and C suggest that the higher the CRF, the lower the risk of CVD and respiratory diseases, but higher risk of MSK diseases (again ignoring the interaction term, a model without interaction should be fitted). The estimates for pulse at rest in models A, B and C are essentially the same.

Authors’ action: We politely suggest that considerations about the consequences of using ratio variables are beyond the scope of the current paper and are already well documented in the literature. Thus, we suggest that adding the considerations above to the manuscript would be superfluous.

Reviewer #3: While many of my comments have been addressed, the revisions appear to have been done rather carelessly, contrasting with the high quality of the previous version of the manuscript. In several places, you claim in your response to have applied (generally minor) changes suggested by me, but on reading the manuscript, you have not. This is very annoying to a reviewer.

Authors’ response: We are very grateful for the careful detailed review, we interpret this as an indication that the reviewer considers the work of interest. We would like to sincerely apologize for not making the revisions as expected by the reviewer, despite our sincere intention to adhere to all the comments. As the only potential explanation, we can say that the short deadline for the revision (3 weeks) combined with an intensive teaching period may have led to suboptimal attention to details.
Nonetheless, my major concern has been mostly addressed and I like the study. If you can address my remaining comments, I think it should be published. I would like to see BMI analysed in SD in the sensitivity analysis, like the other exposures are, and there is an apparent inconsistency in the follow-up period which needs to be resolved. My other comments are matters of language and clarity to the reader.
Authors’ response: Thank you for the encouraging comments. We have detailed our actions below. Thank you very much for noticing the apparent inconsistency in the follow-up time. This was due to a typo (“98” instead of “87”). We apologize for the typo and have corrected it.
Minor issues
Abstract: Colon, not full stop after "Methods and Findings"

Authors’ response and action: Corrected.
Abstract: The abstract still doesn't say which way round the associations with body mass were, doesn't distinguish the three outcomes and doesn't give the CI of the RR. These should be reported the same way as other results in the abstract, e.g. "Higher body mass was associated with higher risk of musculoskeletal (risk ratio X.XX per standard deviation, 95% CI X.XX, X.XX), cardiovascular (x.xx [x.xx, x.xx]) and respiratory diseases (x.xx [x.xx, x.xx])".

Authors’ response and action: We agree, we have corrected.
Author summary: "We found that high body mass as well as tobacco smoking in youth were linked with increased risk for diseases of all the studied diseases later in life" - it would make more sense if you deleted "diseases of".

Authors’ response and action: We agree, we have corrected.
Author summary: Missing full stop on the second bullet point of "What do these findings mean?"

Authors’ response and action: Corrected.
Introduction: The abbreviation OA is still used in paragraph 3 when it has not been properly expanded at first use (in paragraph 2). If you want to use this abbreviation, the text in paragraph 2 should read "…back pain, osteoarthritis (OA), shoulder lesions…". However, the word is written out in full much more often than it is abbreviated to OA, so it would probably be easier to just write it out in full each time.

Authors’ response and action: We agree, we have corrected.
Methods: Should be "…persons alive and resident in Sweden…", not "…persons alive and residents of Sweden…".

Authors’ response and action: Corrected.
Methods: "…chronic pain conditions that have not well understood etiology…" should be "…chronic pain conditions that have poorly understood etiology…".

Authors’ response and action: Corrected.
Methods: I think the naming of supplementary materials is consistently "S Table #" elsewhere, but here you refer to "S1 Table"

Authors’ response and action: Corrected.
Results: Immediately before Table 3, you refer to the cumulative incidence in 1998-2010. I don't see why you go on to say "until year 2010" - isn't this redundant if you have said it's 1998-2010? There's also a bigger problem. The Methods and Figure 1 both suggest that your outcome is the binary diagnosis of the condition in any of the patient groups (inpatients, outpatients, surgery) between 1987 and 2010 (with only inpatient records available for the whole of this period). Why do you then report cumulative incidence in 1998-2010? The cumulative incidence of a disease will depend hugely on the time period over which it is accumulated, so it's important to make this clear.

Authors’ response and action: In response to one of the previous comments, we added the text “diagnosed between 1998 and 2010” and, unfortunately, made a typo. The text should have been “diagnosed between 1987 and 2010”. We agree that with this revision, the last part “until year 2010” is redundant, and we have removed it. The sentence now states:

“The cumulative incidence of musculoskeletal diseases diagnosed between 1987 and 2010 was 20%, cardiovascular 10%, and respiratory diseases 3%.”

Results: Thanks for adding the CI to the paragraph immediately before Table 4. There are some missing spaces in these and a typo in "95%CI 1" to 12%". There are also inconsistent spaces in a few places in "1SD" (I think it should probably have a space).

Authors’ response and action: Corrected. We have included spaces between 1 and SD where they were missing. The 1” should have been 1%.
Table 3: You said that you have added a decimal place of precision to some of the means in Table 3 as I requested, but this has not been done. You have also not added the units of pulse rate.

Authors’ response and action: We apologize for the unintentional omission. We have now added the units of pulse at rest (beats per minute) and added one decimal to the descriptive values of body mass, body mass index, pulse at rest and hematocrit.

Figure S1: This is great for showing the (minimal) effect of different adjustment sets. One small point which I missed first time round is that ratios are conventionally plotted on a log scale. That is; you plot log(RR) on a linear scale, but label tick marks with RR (so if your tick marks are at regular intervals, they get closer together at higher values). This applies to your main Figure 2 as well as Figure S1. You've also missed a "d" in "fully ajusted" in the legend. The fact that the choice of adjustment set makes little difference to the estimates is of value to the reader, so in the main manuscript I suggest writing something like "Differences in adjustment variables made little difference to the estimates (S Figure 1)" instead of just "The magnitude of change in the estimates due to adjustments can be seen in S Figure 1".

Authors’ response: We agree.

Authors’ action. We have changed the x-axis scale to log in Figure 2 and S Figure 1. We have corrected the typo in the legend of S Figure 1. We have revised the text in the results section, but added “for physical health measurements”, as the magnitude changed more for the lifestyle variables, especially for estimates of associations with cardiovascular and respiratory diseases.

“Differences in adjustment variables made little difference to most of the estimates for physical health measurements.(S Figure 1)”
Figure 1: The second sentence isn't grammatically correct; it should read something like "All exposures were included in the model and they were also adjusted for parental education and occupation". I think some explanation is needed of the points either side of the mean; you could perhaps expand the sentence to read "The middle value on the x-axis corresponds to the mean value (or mode for cardiorespiratory fitness) in the study sample and exposures are plotted over a range of two SD either side of this". The cumulative incidences at the means of these exposures are the same as the 20%, 10% and 3% reported in the third paragraph of the results, but here you refer to them as covering 1987-2010 (which is also what the methods suggest) whereas the earlier report says they are 1998-2010. Please check this.

Authors’ response and action: We have revised the legend to:

“Figure 1. Fitted incidence proportion by measures of physical health. Marginal estimates from the adjusted Poisson regression model. All exposures were included in the model and they were also adjusted for parental education and occupation. The middle value on the x-axis corresponds to the mean value (all exposures apart from cardiorespiratory fitness) in the study sample and exposures are plotted over a range of two SD on either side of this. For cardiorespiratory fitness, the values correspond to mid points of the intervals (where the middle value on the x-axis is the mode). Incidence proportion refers to the proportion of persons with diseases diagnosed during the follow-up period from 1987 to 2010.”

The "1998” was a typo and has now been corrected.

Finally, I think the colours are better than they were but I suspect some colour-blind people would still struggle with them, as well as anyone who printed the paper
greyscale. Why not use different styles of point or line to avoid this problem, as you have in Figure 2?

Authors’ response and action: We have now used different symbols for the different outcomes also in the figure 1.
Discussion: It should be "Increased pain sensitivity as a consequence of headache...", not "Increased pain sensitivity as consequence of headache..."

Authors’ response and action: Corrected.
Discussion: There's an unfinished sentence at the end of the penultimate paragraph: "Headache, especially frequent,". Did you mean to continue this or delete it?

Authors’ response and action: Thank you for noticing, this has been deleted.
Data statement: Should be "national Swedish registers", not "national Swedish registered". Also "included in the supplementary file", not "included in supplementary file".

Authors’ response and action: Corrected.
Conflicts of interest: Should be "AK acts..." not "AK act...".

Authors’ response and action: Corrected.
S Table 3: BMI has been included here, but there's no results for it. It would be useful if the adjusted and unadjusted BMI models could be compared.

Authors’ response and action: Thank you for noticing this unintentional omission. We have included the estimates. We present them now per 1SD of BMI.
S Table 5: BMI should be analysed in SD, like the other exposures (not per 5 units). This shouldn't change the (minimal) effect of adjusting for BMI instead of height and mass, on the other exposures, but it makes it much easier to compare the estimates for BMI itself with those for mass. I found myself making comparisons on the basis of guesswork - I might expect BMI in this sample to have a SD of about 3 units, which would make the RR per SD about 1.13 - very close to your result for body mass. But if you analyse BMI in SD, readers will be able to see this similarity much more clearly.

Authors’ response and action: We agree, we have revised. We have additionally included BMI in the Table 3 to provide descriptive data also on this exposure used in the sensitivity analyses.
Supplementary code: This looks a bit incomplete - could you include the code used for the other adjustments sets, the sensitivity analyses, etc? Also, you label a variable called "outocme" (not "outcome"); this would cause an error message if no such variable exists.

Authors’ response and action: We agree. We have expanded the code to include univariable analyses, analyses adjusted for body size, and some sensitivity analyses and analysis of specific MSK outcomes and corrected the typo in “outcome”.
