# Supplementary material for: Physical health in young males and risk of chronic musculoskeletal, cardiovascular, and respiratory diseases by middle age: A population-based cohort study
Source: PLoS Med. 2025 Jan 21;22(1):e1004517. doi: 10.1371/journal.pmed.1004517 (PMC11793790; doi:10.1371/journal.pmed.1004517)
Supplement: Attachment — Submitted filename: Point_to_point_response_Turkiewicz_20241122_2.docx [file pmed.1004517.s005.docx]

We would like to sincerely thank all the reviewers for their careful review, relevant comments and interest in our work. We provide a point-by-point response below. We hope that the reviewers will find the revised work acceptable.

Comments from the editorial team:

We are happy for you to keep the primary analysis as it is. We ask that you (1) provide additional text in the paper explaining your decision not to index, and (2) provide this as a sensitivity analysis, as outlined/requested by the reviewer.

Authors’ response and action: Thank you for the careful review and guidance. We have performed a sensitivity analysis with indexed CRF, provided the results in appendix, included explanations in the methods section, added main estimates in the main text results section and included a short text in the discussion section. Please see description of the specific changes in our response to reviewer #2 below.

-----------------------------------------------------------
Comments from the reviewers:

Reviewer #2: Thank you for responding to my comment.  The key issue here is power outputs in Watts is not the correct measure of CRF.  Calling this variable CRF is not correct. The correct variable for CRF here is W/kg. CRF is generally used to provide an assessment of physical performance. For example in the classic Aerobics Centre Longitudinal Study papers examining the association between CRF and health outcomes, the CRF variable used was treadmill running time as measured in an incremental test.  This variable would correspond to W/kg. VO2max in ml/kg/min, but not in l/min, is a strong predictor of running performance.  Thus this issue here is that the exposure variable should be measured in W/kg, not W. Virtually all of the epidemiological literature on the association between CRF and health outcomes will express CRF in this way (i.e. running performance, VO2max in ml/kg/min, VO2 max in METS (where 1 MET is 3.5 ml/kg/min), or in W/kg). Including body mass separately in
the model and then saying that the W value represents CRF is not correct.  If you follow the authors logic to its conclusion, then almost 50 years of epidemiological research on CRF and health outcomes (and even longer examining CRF and exercise performance) is wrong, as is the widely used concept of using METS to express fitness and exercise intensity. It is bold to assert that your approach is correct, and the rest of the field is wrong, and I don't think that the authors have made a convincing argument to that effect.  Model A is the correct model to use here, and, as I suspected, when you express CRF in terms of W/kg, a higher level of CRF is associated with lower (rather than higher) risk of MSK disease (as it does for CVD and respiratory disease), and this is true in models with and without resting heart rate.  This is the expected finding here and has biological plausibility.  The original finding that CRF is positively associated with risk of MSK diseases is an
artifact of expressing CRF using inappropriate units.  The findings are still worthy of publication with the new (correct) analysis, as presented in model A, with appropriate reinterpration of the implications. Please could the authors reanalyse the data along these lines and also revise the discussion as appropriate.

I understand that this involves a substantial amount of work and that the authors are reluctant to make changes that alter the paper's key message. However, I do feel that this is important to make this correction, and that this paper (with the appropriate data analysis and interpretation) can make an important contribution to the literature.

Authors’ response and actions: Thank you for the careful review. In accordance with the guidance from the editorial team, we have kept our main analysis as is, but included a new appendix with sensitivity analyses using CRF expressed as W/kg. We made following specific changes:

a) We have explained what we mean when using the term “cardiovascular fitness” (methods, section “Exposures”, after description of the cycle ergometer test)

“Thus, we use the term “cardiovascular fitness” to denote this variable (unit Watt).[19]”

b) We have added a new text in the statistical methods section describing the sensitivity analyses.

“Prompted by comments from reviewers we included several sensitivity analyses. We included results adjusted for BMI (instead of body mass and height), and adjusted for pulse at rest or cardiorespiratory fitness, but not both in the same model. Further, as cardiorespiratory fitness is often expressed normalized by body mass (W/kg) we fitted models with such exposure (S2 Results – cardiorespiratory fitness). We did not use this variable in our main analyses due to known interpretational problems when using ratio variable, which is a statistical interaction [22,26]. We fitted models without and with main effects of both variables.”

c) We have added new text in the results section to provide the new estimate also in the main text.

“The coefficient for cardiorespiratory fitness normalized by body mass vs musculoskeletal disease, measuring the relationship of both variables with the disease risk, was 0.96 (0.93, 0.98) (S2 Results – cardiorespiratory fitness).”

d) We have added a new appendix with results from sensitivity analyses – file “S2 Results – cardiorespiratory fitness”. We provide the table with adjusted results below for convenience.

S Table 10. The adjusted risk ratios (RR) with 95% confidence intervals (CI) for associations between cardiorespiratory fitness normalized by body mass (W/kg), with and without main effects of the interaction variables.

| Exposure and model | **Musculoskeletal** | | **Cardiovascular** | | **Respiratory** | |
| --- | --- | --- | --- | --- | --- | --- |
|  | RR | 95%CI | RR | 95%CI | RR | 95%CI |
| Model A |  |  |  |  |  |  |
| CRF/body mass (W/kg) | 0.96 | [0.93,0.98] | 0.86 | [0.82,0.89] | 0.88 | [0.81,0.94] |
| Model B |  |  |  |  |  |  |
| CRF/body mass (W/kg) | 0.96 | [0.92,1.00] | 0.95 | [0.89,1.01] | 0.93 | [0.83,1.04] |
| CRF (W) | 1.05 | [0.99,1.11] | 0.88 | [0.82,0.95] | 0.83 | [0.72,0.96] |
| Inverse of body mass (1/kg) | 0.88 | [0.85,0.91] | 0.79 | [0.76,0.83] | 0.86 | [0.78,0.94] |

e) We included new text in the discussion section (at the end of the paragraph about cardiorespiratory fitness) :

“Another important factor to consider is that in the main analyses we used cardiorespiratory fitness measure from physical work capacity test, not normalized by body mass. Such normalized cardiorespiratory fitness is often a good predictor of performance, but may be less suitable for describing epidemiological associations, as it represents both relationship with cardiorespiratory fitness and inverse of body mass. When the two relations are in opposite directions (as in our data), such a combined estimate may be difficult to interpret.[19,22]”

These changes have led to inclusion of two new references:

19. Raghuveer G, Hartz J, Lubans DR, Takken T, Wiltz JL, Mietus-Snyder M, et al. Cardiorespiratory Fitness in Youth: An Important Marker of Health: A Scientific Statement From the American Heart Association. Circulation. 2020 Aug 18;142(7):e101–18.

26. Packard GC, Boardman TJ. The use of percentages and size-specific indices to normalize physiological data for variation in body size: wasted time, wasted effort? Comp Biochem Physiol A Mol Integr Physiol. 1999 Jan 1;122(1):37–44.

Reviewer #3: Thank you for the comprehensive response. You have addressed all the points in my most recent review. There was one thing which I missed previously (sorry) but which I think should be corrected. In most of the supplementary tables and in Table 4, the variable "Stomach problems" has four levels; often, sometimes, occasionally and never (reference category). In S Tables 3-5, the reference category is omitted - this doesn't leave out any important information but I think it should be added for clarity and for consistency with the other categorical variables. Perhaps more importantly in S Table 3 the "occasionally" category is also missing - I'm guessing this is either an accidental omission which should be corrected, or for some reason categories were merged/left out of this analysis (in which case this should be explained in the footnotes). I haven't checked through all the variables in all the tables, but please can you make sure that they are complete?

Authors’ response and actions: Thank you for the careful review. The omission of “occasionally” category was unintentional, it is now included. We have also included rows for reference categories in S Table 5, S Table 4 and S Table 3. We have checked the tables for completeness of all variable categories and added reference category for “Drug use” in S Tables 7 and 8.

There is also a missing full stop in the revised notes on Figure 1.

Authors’ response and action: Corrected.

It's for these minor reasons that I've responded with "Minor Revisions" rather than "Accept", but they should be easily sorted out between authors and editor and I don't think I need to see another draft. I look forward to seeing the published article.

Authors’ response and actions: Thank you for the positive feedback.
