# Supplementary material for: Physical health in young males and risk of chronic musculoskeletal, cardiovascular, and respiratory diseases by middle age: A population-based cohort study
Source: PLoS Med. 2025 Jan 21;22(1):e1004517. doi: 10.1371/journal.pmed.1004517 (PMC11793790; doi:10.1371/journal.pmed.1004517)
Supplement: Attachment — Submitted filename: Turkiewicz_young_men_point_by_point_20241217_v2.docx [file pmed.1004517.s007.docx]

We would like to thank the expert reviewers for their positive feedback and the editorial team for their careful supporting guidance for this manuscript and for the positive evaluation. We have made all the required changes and provide a point-by-point response below in blue.

Comments from Reviewers:

Reviewer #2: Thank you for including the sensitivity analysis. I understand the argument that maximum power in Watts and body mass have associations with MSD in opposite directions. However maximum power is Watts is not the appropriate measure of cardiorespiratory fitness, it is in fact a measure of cardiorespiratory fitness x body mass. This is because cardioresiratory fitness is related to your ability to move your own body (and therefore your own body mass), not your ability to move an external load.  Hence normalisation to body mass is needed. I would be content with the paper if the variable that is currently referred to as "cardiorespiratory fitness" is renamed "maximal power output" (or similar wording). This is more technically correct and gets round the authors' concerns about using a ratio variable.  Would the authors be able to make this minor change in terminology.  I would be happy to accept the paper if they could do this.

Authors’ response and action: Thank you for the positive feedback and careful review. In line with the guidance from the editorial team (please see item 4 “Request from editors” below) we have left the terminology as is, but further explained in the methods section that when using the term “cardiorespiratory fitness” we mean the maximal power output from the physical work capacity test performed on a bicycle ergometer.


------------------------------------------------------------
Requests from Editors:

GENERAL

1) Please note that we require authors to add line and page numbers for review purposes. We have added line numbers to the clean version document (Turkiewicz_Physical health_young men_main_rev20241128_clean) and attached the document for your convenience.

Authors’ action: Thank you, we have done all following revision in this provided version.

2) Please note that references to tables and/or figures (including supplementary material) should be placed before punctuation. This also applies to citations.

Authors’ action: We have gone through the main text and appendix and corrected all instances found.

3) There appear to be some instances, e.g. l.289, where you use 'cardiovascular fitness' instead of 'cardiorespiratory fitness'. Please check (throughout) and revise if necessary.

Authors’ action: Thank you for noticing this error. We have revised to “cardiorespiratory fitness”.

4) We have discussed Reviewer #4's final request with the Academic Editor to change the wording from "cardiorespiratory fitness" to "maximal power output" (or similar). The Editorial Team and the Academic Editor suggest adding an additional line in the Methods that mentions maximal power output, but we will not ask you to replace the term "cardiorespiratory fitness" throughout the manuscript.

Authors’ action: We have added the phrase “maximal power output” when defining the CRF exposure, the full sentences now read:

“The measurements were done according to standardized protocols [18]. Cardiorespiratory fitness (physical work capacity) was tested by means of the maximal work test, where the tested person is required to work, until reaching the point of exhaustion, on a bicycle ergometer with an invariable, heavy load. The load was chosen individually with respect to constitution and prior history of physical activity. Thus, we use the term “cardiorespiratory fitness” to denote this variable (maximal power output, unit Watt) [20].”

TITLE

Please revise your title according to PLOS Medicine's style. Your title must be nondeclarative and not a question. It should begin with main concept if possible. "Effect of" should be used only if causality can be inferred, i.e., for an RCT. Please place the study design ("A randomized controlled trial," "A retrospective study," "A modelling study," etc.) in the subtitle (ie, after a colon).

Authors’ action: We have revised to:

Physical health in young males and risk of chronic musculoskeletal, cardiovascular and respiratory diseases by middle age: A population-based cohort study

ABSTRACT

1) l.3: Please replace ‘elderly’ with ‘older’. Please revise throughout.

Authors’ action: We have replaced all occurrences of “elderly” with “older”.

2) l.12: Please define ‘GEE’ at first use.

Authors’ action: We have spelled out GEE in abstract methods and findings.

3) l.14ff: For all numerical results, please re-iterate the statistical information for each pair of parentheses. Fo example: “We found that higher body mass was associated with higher risk of musculoskeletal (risk ratio [RR] per 1 standard deviation [SD] 1.12 [95% confidence interval 1.09, 1.16]), cardiovascular (RR 1.22 [95% CI (1.17,1.27)] per 1 SD)…” Please revise throughout for the Abstract and the main text for consistency.

Authors’ action: We have revised to include RR, 95% CI and “per 1 SD” when reporting relevant estimates. We tried to avoid including too many parentheses and brackets to keep readability. In the main text, we have replaced the risk ratios expressed as percentages to ratio estimates for consistency with the abstract and tables.

4) l.16ff: Please note that you have used interpuncts for the next two brackets. Please revise.

Authors’ action: Thank you for noticing. We have replaced them with points.

5) ll.22-24, please revise for clarity, for example: “Self-reported headache (category often compared to never) was associated with musculoskeletal diseases (RR 1.38 [95% CI (1.21,1.58) per 1 SD] and cardiovascular diseases (RR 1.29 [95% CI (1.07,1.56) per 1 SD], but not with respiratory diseases (RR 1.13 [95% CI (0.79,1.60) per 1 SD].”

Authors’ action: We have revised. Given the values included in the CI for respiratory disease we think stating “no association” would be suboptimal, and we report it as “inconclusive”. We hope this is acceptable. The sentence now reads:

“Self-reported headache (category often compared to never) was associated with musculoskeletal diseases (RR 1.38 [95% CI 1.21, 1.58]) and cardiovascular diseases (RR 1.29 [95% CI 1.07, 1.56]), but had inconclusive association with respiratory diseases (RR 1.13 [95% CI 0.79, 1.60]).”

6) In the last sentence of the Abstract Methods and Findings section, please describe the main limitation(s) of the study's methodology.

Authors’ action. We have included the following sentence:

“The main limitations include lack of adjustment for genetic factors and environmental exposures from childhood, and that the register data were available for males only.”

7) Abstract Conclusions: Please use the past tense when describing results, i.e. “While high body mass was a risk factor for all three studied groups of diseases, high cardiorespiratory fitness and high muscle strength in youth were associated with increased risk of musculoskeletal disease in middle age.”
Authors’ action: We have revised as requested. We have also revised similar statements in the main text to past tense for consistency.

8) Please ensure that all numbers presented in the abstract are present and identical to numbers presented in the main manuscript text.

Authors’ action: We have added the estimates for cardiorespiratory fitness and osteoarthritis to the main text. All other estimates were already reported in main manuscript text or tables. We have also checked that estimates in the abstract and main text are identical. To make it more explicit, we replaced two sets of estimates in the main text from being expressed as percentages to being expressed as ratio estimates (the same as in the abstract).

9) Please include the important dependent variables that are adjusted for in the analyses.

Authors’ action: We clarified that all exposures were included in one model (and thus mutually adjusted).

10) We suggest detailing the specific diseases included in the three main groups in the abstract, as there aren't too many. For example, on lines 10-11: We followed the participants through the Swedish National Patient Register for incidence of common musculoskeletal (Shoulder lesions, myalgia, osteoarthritis, joint pain, back pain), cardiovascular (Ischaemic heart disease, atrial fibrillation), and respiratory diseases (Asthma, chronic obstructive pulmonary disease, bronchitis).

Authors’ action: We have revised as requested.

AUTHOR SUMMARY

Please change the final bullet point of 'What Do These Findings Mean?' to: The main limitation is that based on the data source, we were only able to include males in the study.

Authors’ action: We have revised as requested.

INTRODUCTION

1) If there has been a systematic review of the evidence related to your study (or you have conducted one), please refer to and reference that review and indicate whether it supports the need for your study.

Authors’ action: To the best of our knowledge, there is no systematic review of risk factors in youth and future development of musculoskeletal diseases.

2) l.71: Please refer to low or middle income countries rather than "developing countries" or "the Global South".

Authors’ action: We have removed “in developed countries”, as cardiovascular disease is highly prevalent worldwide.

3) ll.72-74: Please provide references.

Authors’ action: We have included two references (one of them is a new reference) and added the word “extensive” prior to alcohol consumption.

METHODS AND RESULTS

1) Table 4: Do the values that show 1.00 represent the reference for the exposure in question? If so, we suggest you clarify this.

Authors’ action: We have added “(reference)” to the description of these categories in Table 4.

2) Figure 1: Please list all exposures in the figure description and define musculoskeletal, cardiovascular and respiratory. Please note that you talk about “exposures” in table 3 and in table 4, so it is important to clarify which set of exposures you refer to. Also, please define ‘SD’ and ‘CI’.

Authors’ action: We have spelled out standard deviation and added a list of diseases included in each of the three disease groups and definition of CI. We listed all exposures included in the model.

3) Figure 2: Please define ‘CI’.

Authors’ action: Done.

4) Please briefly mention the results form the sensitivity analyses in the main text (S Table 9 and 10). In general, please make sure that findings from any sensitivity analyses are briefly explained, e.g. whether they support the findings of the main analyses.

Authors’ action: These results were already mentioned in line 265. We have now changed the names of supplementary tables, figures and text according to the instructions below, which means that we now reference these appendix tables explicitly. We checked that all appendix material is referenced in the main text and added a new sentence about results from multiply imputed data to the results text. We discuss the interpretation of sensitivity analyses in Previous Tables 9 and 10 (renamed to Tables I and J) in the discussion section.

5) l.247ff: We have noticed that you do not present findings on all exposures of interest, and that you report findings selectively. For example, findings on height, blood pressure, resting pulse, haematocrit, drug use and alcohol intake are not mentioned at all. We feel that for a balanced presentation, even if no associations were found between the exposures and the three disease groups, this should be clearly stated in the results section. Please revise (throughout).

Authors’ action: We do present all findings in Table 4 and figures (and related appendix material). We understand that it is expected that we would mention all results in the text itself as well. We have added a new text about height just after body mass. We added a new paragraph to mention results for blood pressure, resting pulse and hematocrit. We added results on drug use and alcohol intake in the paragraph about lifestyle factors. We politely refrain from repeating all estimates from the Table 4 in the results text.

6) ll.272-274, “Surprisingly, headache seems to present an association with a “dose-response” pattern with both musculoskeletal and cardiovascular diseases, even though the confidence intervals are rather wide.” – please re-phrase and avoid describing the associations as a “dose-response” pattern.

Authors’ action: We have revised without using “dose-response”.

7) ll.248-250, “The associations between the exposures and the different outcomes (disease groups) differ both in magnitude and direction, both in unadjusted (S Table 3) and adjusted analyses (Table 4, S Tables 4, 5, 6).” – We suggest that this should be explained in more detail. The description “differ both in magnitude and direction” is rather vague.

Authors’ action: We describe all the results in the following text, where we report both direction (increase or decrease in risk) and magnitude (the size of the associations). We have revised this sentence to just include references to the specific tables with results, without statements about “magnitude and direction”. The text now reads:

“The unadjusted associations between the exposures and the different outcomes (disease groups) are presented in Table C in S1 Supporting Information, the adjusted associations in Table 4 and results from sensitivity analyses in Tables D, E and F in S1 Supporting Information. Differences in adjustment variables made little difference to most of the estimates for physical health measurements (Fig A in S1 Supporting Information) and the results from sensitivity analyses were similar to the main findings (Tables D, E and F in S1 Supporting Information).”

8) ll.287-289: “Most exposures show the same direction and similar magnitude of the associations with all included musculoskeletal diseases.(Figure 2) However, the weakest (or inconclusive) associations are with joint pain and myalgia” – Similar to earlier, these statements seem rather vague and do not really say much about the actual results. It could even be argued that they do not reflect the results shown in the figure. Please revise for accuracy.

Authors’ action: We have removed the sentence about direction and magnitude. The first sentence of the paragraph now reads:

“Higher body mass was associated with all included specific musculoskeletal diseases (Figure 2, Table G in S1 Supporting Information).”

We have added sentences explaining the statements about myalgia and joint pain:

“The weakest (or inconclusive) associations were with joint pain and myalgia. For myalgia, all estimates apart from those for body mass and hematocrit were close to 1. All confidence intervals for estimates of associations with joint pain were wide.”

We have revised the rest of the paragraph to include more estimates. We do not list all estimates from Table G, but rather provide examples with referral to the supplementary table. The table contains all estimates.

9) ll.289-289, “However, the weakest (or inconclusive) associations are with joint pain and myalgia.” – please elaborate.

Authors’ action: We have added new sentences.

“The weakest (or inconclusive) associations were with joint pain and myalgia. For myalgia, all estimates apart from those for body mass and hematocrit were close to 1. All confidence intervals for estimates of associations with joint pain were wide.”

10) ll.289-291, “Higher cardiovascular fitness and lower pulse at rest, after adjusting for other exposures, seem to be associated with higher risk of osteoarthritis, while not as clearly with other included musculoskeletal diseases.” – The risk ratio for pulse at rest in the OA group is 0.9 [0.87,0.94], which seems to indicate a lower risk of OA. Please clarify.

Authors’ action: Yes, the risk ratio for association between pulse at rest and OA is 0.9, which means that higher pulse at rest is associated with lower risk of disease. Conversely, lower pulse at rest is associated with higher risk of disease. We have revised to avoid confusion. The revised sentences read:

“Higher cardiorespiratory fitness, after adjusting for other exposures, seemed to be associated with higher risk of osteoarthritis (RR 1.23 [95% CI 1.15, 1.32] per 1 SD), while not as clearly with other included musculoskeletal diseases. Higher pulse at rest was associated with lower risk of osteoarthritis (RR 0.91 [95% CI 0.87, 0.94] per 1 SD), but not with back pain.”

11) l.293: “for other specific diseases.” – please specify.

Authors’ action: We have revised to:

“while the estimates were smaller in magnitude for osteoarthritis, myalgia or joint pain (Figure 2, Table G in S1 Supporting Information).”

12) ll.293-295, “Alcohol use and smoking, as well as self-reported health measures have generally similar associations with all included musculoskeletal diseases.” – What are these associations? Again, please provide more details and do not report on exposures selectively.

Authors’ action: All results are presented in S Table 7 (now renamed to Table G) and Figure 2. We do reference to this table in the results text, but we do not repeat all estimates in the text. We have expanded this section to provide examples of the estimates. Given the number of exposures and musculoskeletal conditions, it would be difficult to list all estimates in text.

“Alcohol use and smoking, as well as self-reported health measures had generally similar associations with all included musculoskeletal diseases (Table G in S1 Supporting Information). For example, smoking 11+ cigarettes per day compared to non-smoker had risk ratios of 1.20 (95% CI 1.07, 1.34) with myalgia, 1.03 (95% CI 0.94, 1.12) with osteoarthritis, 1.19 (95% CI 1.02, 1.39) with shoulder disease, 1.30 (95% CI 1.15, 1.47) with back pain and 1.12 (95% CI 0.93, 1.35) with joint pain.”

DISCUSSION

General guidance: Please present and organize the Discussion as follows: a short, clear summary of the article's findings; what the study adds to existing research and where and why the results may differ from previous research; strengths and limitations of the study; implications and next steps for research, clinical practice, and/or public policy; one-paragraph conclusion.

Authors’ response: We believe our discussion section generally follows the guidance above, but we have moved sentences related to “implications and next steps for research” to a separate paragraph before discussion. To improve readability, we added one new general sentence. We hope this is acceptable.

1) l.309: Please temper claims of primacy of results by stating, "to our knowledge" or something similar. Please revise throughout (e.g line 394).

Authors’ action: We have included “to our knowledge” in previous line 309, rephrased “have not been studied previously” to “are understudied” and removed the term “novel” from the conclusions. We have removed “one of a kind” from the first paragraph.

2) Please remove any subheadings. The conclusion should be a continuous part of the discussion.

Authors’ action: Done.

REFERENCES

Where website addresses are cited, please use the word ‘accessed’ when specifying the date of access (e.g. [accessed: 12/06/2024]).

Authors’ action: We have revised “cited” to “accessed:” in the reference list where referring to the National Patient Register.

SUPPLEMENTARY MATERIAL

1) Please note that all supplementary files should be references in the main text (e.g. S Table 2 does not seem to be referenced). Please check and revise.

Authors’ action: We have included a new sentence and reference to this table (now denoted Table B in S1 Supporting Information). We also included a new sentence with the reference to Table H in S1 Supporting Information. We checked that all supplementary material (including methods) is referenced in the text.

2) In the published article, supporting information files are accessed only through a hyperlink attached to the captions. For this reason, you must list captions at the end of your manuscript file. You may include a caption within the supporting information file itself, as long as that caption is also provided in the manuscript file. Do not submit a separate caption file.

When SI files are contained with a single file:
Please label the file as ‘S1 Supporting Information’.
Please apply alphabetical labelling to each table and figure contained within the S1 file. For example, ‘Fig A’ to ‘Fig Z’ and ‘Table A’ to ‘Table Z’.
Plain text does not need to be labelled and can just be given a title as necessary. For example, ‘Statistical Analysis Plan’.
Please cite tables/figures as ‘Fig A in S1 Supporting Information’ and/or ‘Table A in S1 Supporting Information’, for example.
Please cite plain text as, ‘Statistical Analysis Plan in S1 Supporting Information’, for example.

Authors’ action: We have compiled all supplementary material (apart from S1 Checklist) in one file and renamed all tables, figures, text accordingly and revised the captions in the main text. In the main text, we provide caption “S1 Checklist” and “S1 Supporting Information”. In the S1 Supporting Information file, we included a list of contents in the beginning of the document. We do reference each item in the S1 Supporting Information file in the manuscript.

When SI files are uploaded as separate files:
Please label tables as ‘S1 Table’ (so on) and figures as ‘S1 Fig’ (and so on).
Any additional documents (protocols/analysis plans etc.) can be labelled as ‘S1 Protocol’, for example. Please cite items as exactly as labelled.

Authors’ action: Not applicable, we combined supplementary material in one file.

------------------------------------------------------------
General Editorial Requests

1) We ask every co-author listed on the manuscript to fill in a contributing author statement. If any of the co-authors have not filled in the statement, we will remind them to do so when the paper is revised. If all statements are not completed in a timely fashion this could hold up the re-review process. Should there be a problem getting one of your co-authors to fill in a statement we will be in contact. YOU MUST NOT ADD OR REMOVE AUTHORS UNLESS YOU HAVE ALERTED THE EDITOR HANDLING THE MANUSCRIPT TO THE CHANGE AND THEY SPECIFICALLY HAVE AGREED TO IT.

Authors’ action: We understand.

2) Please ensure that the paper adheres to the PLOS Data Availability Policy (see <http://journals.plos.org/plosmedicine/s/data-availability)>, which requires that all data underlying the study's findings be provided in a repository or as Supporting Information. For data residing with a third party, authors are required to provide instructions with contact information for obtaining the data. PLOS journals do not allow statements supported by "data not shown" or "unpublished results." For such statements, authors must provide supporting data or cite public sources that include it.

Authors’ action: We understand, there is a data availability statement included in our manuscript.

3) Please review your reference list to ensure that it is complete and correct. If you have cited papers that have been retracted, please include the rationale for doing so in the manuscript text, or remove these references and replace them with relevant current references. Any changes to the reference list should be mentioned in the rebuttal letter that accompanies your revised manuscript.

Authors’ action: The only changes in the reference list made are in response to guidance above. I.e., we included one new reference (and listed one existing in an additional place) as reported above and we have revised “cited” to “accessed:” for one online resource.
